# A Novel Data-Driven Boolean Model for Genetic Regulatory Networks

**DOI:** 10.3389/fphys.2018.01328

**Published:** 2018-09-25

**Authors:** Leshi Chen, Don Kulasiri, Sandhya Samarasinghe

**Affiliations:** ^1^Computational Systems Biology Laboratory, Centre for Advanced Computational Solutions, Lincoln University, Lincoln, New Zealand; ^2^Integrated Systems Modelling Group, Centre for Advanced Computational Solutions, Lincoln University, Lincoln, New Zealand

**Keywords:** boolean modeling, boolean network, time series data, network inference, data-driven boolean modeling, fundamental boolean model, fundamental boolean networks, orchard cube

## Abstract

A Boolean model is a simple, discrete and dynamic model without the need to consider the effects at the intermediate levels. However, little effort has been made into constructing activation, inhibition, and protein decay networks, which could indicate the direct roles of a gene (or its synthesized protein) as an activator or inhibitor of a target gene. Therefore, we propose to focus on the general Boolean functions at the subfunction level taking into account the effectiveness of protein decay, and further split the subfunctions into the activation and inhibition domains. As a consequence, we developed a novel data-driven Boolean model; namely, the Fundamental Boolean Model (FBM), to draw insights into gene activation, inhibition, and protein decay. This novel Boolean model provides an intuitive definition of activation and inhibition pathways and includes mechanisms to handle protein decay issues. To prove the concept of the novel model, we implemented a platform using R language, called *FBNNet*. Our experimental results show that the proposed FBM could explicitly display the internal connections of the mammalian cell cycle between genes separated into the connection types of activation, inhibition and protein decay. Moreover, the method we proposed to infer the gene regulatory networks for the novel Boolean model can be run in parallel and; hence, the computation cost is affordable. Finally, the novel Boolean model and related Fundamental Boolean Networks (FBNs) could show significant trajectories in genes to reveal how genes regulated each other over a given period. This new feature could facilitate further research on drug interventions to detect the side effects of a newly-proposed drug.

## Background

DNA carries the genetic information that governs life, death and the reproduction of living organisms. A gene is a fragment of DNA that codes for one protein, the fundamental unit of cellular functions. Gene expression is the process whereby a gene initially transcripts into mRNA, then mRNA translates it into the protein (Albert, 2004). The central dogma of cellular functions mainly relies on the coordinated interactions between genes, RNAs, and proteins that form the foundation of genetic regulatory networks (GRNs). Within GRNs, activators and inhibitors are very important because they control the patterns of gene expression that regulate cellular functions (Shmulevich et al., 2002a). An activator is a transcription factor (TF) type of protein that increases the concentration and activity of a protein through direct binding to the protein or the promoter sites of its genes. The process is called gene activation (Saboury, 2009), while an inhibitor is a repressor that decreases the concentration and activity of the protein. The process is called gene inhibition (Saboury, 2009). Inhibition has been extensively analyzed because inhibitors can be used as pharmaceutical agents in human and veterinary medicine as well as in herbicides and pesticides (Fontes et al., 2000; Saboury, 2009).

In systems biology, studying the relationships between the functional status of proteins and gene expression patterns in GRNs in a holistic manner is critical to understand the nature of cellular functions as well as their dysfunctions; for example, in triggering diseases (Shmulevich et al., 2002a). However, the identification of GRNs based on the current experimental methods is usually inefficient due to the lack of reproducibility for a large number of genes usually involved in complex GRNs (Liu et al., 2016). Powered by biotechnologies, such as Affymetrix™ microarray technology, enormous amount of high-throughput genetic data have been generated, enabling reverse engineering of unknown regulatory networks revealing the relationships among the functional genes as in mammalian cell cycle (Fauré et al., 2006; Ruz et al., 2014) and leukemia (Saez-Rodriguez et al., 2007; Wittmann et al., 2009; Hwang and Lee, 2010; Saadatpour et al., 2011, 2013; Zañudo and Albert, 2013; Campbell and Albert, 2014). It is evident from these examples that it is a significant challenge to analyse the massive data sets to understand the coordinated interactions among genes.

Boolean modeling is the simplest model for GRNs without the need to consider any effects at the intermediate levels (Liang et al., 1998; Tušek and Kurtanjek, 2012; Abou-Jaoude et al., 2016; Barberis et al., 2017; Traynard et al., 2017). This modeling was initially introduced by Kauffman (Kauffman, 1969; Kauffman et al., 2003) in 1969 following the discovery of the first gene regulatory mechanisms in bacteria (Jacob and Monod, 1961). Since then, Boolean networks have been intensively used for modeling gene regulation.

A Boolean model is constructed using Boolean variables in either of two binary states - *On* (1) or *Off* (0) - that represent gene activation or inhibition, respectively. Each variable represents a gene included in the GRNs with its next state affected by a Boolean function. A Boolean function, denoted by *f*, is a logic rule that gives a Boolean value, of 0 or 1, as an output based on the logic calculation of the Boolean input, as defined in Equation (1).

(1) $$\begin{array}{r} f:\left\{0,1\right\}\to \left\{0,1\right\} \end{array}$$

**Figure 1:**
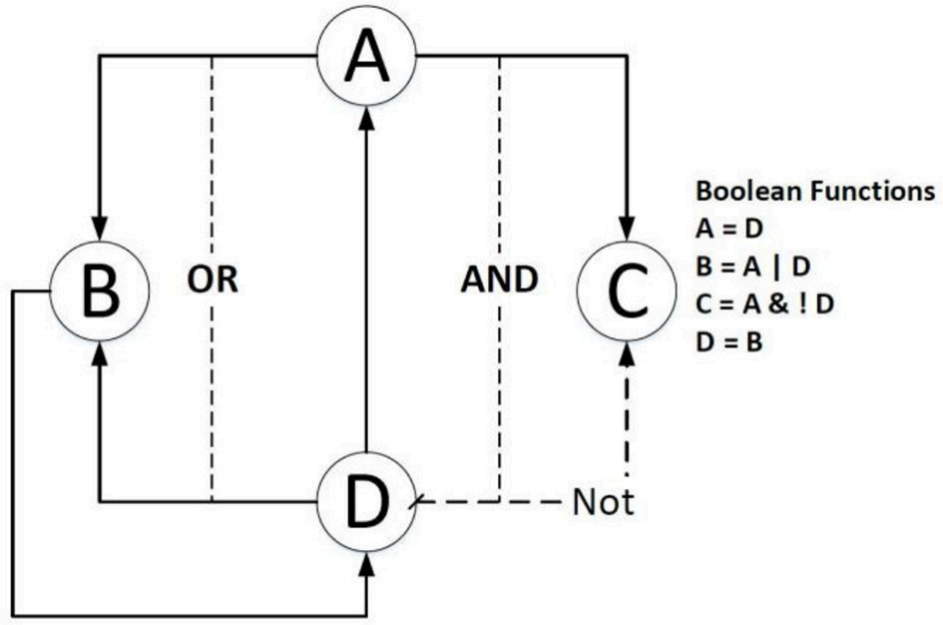
An example of a simple Boolean Network. The right side lists the Boolean functions of the example; the dashed line means the source gene is expected to be inhibited; the solid line indicates that the source gene is expected to be activated.

Figure 1 shows an example of a Boolean network in which gene *B* is dependent on the activation of gene *A* or gene *D*, and gene *C* is related to the activation of gene *A* and the inhibition of gene *D*.

The basic premise of the Boolean network is that the genes exhibit switch-like behavior during the regulation of their functional states. The switch-like behavior ensures the movement of a GRN from one state to another (Shmulevich et al., 2002a; Shmulevich and Dougherty, 2005; Tušek and Kurtanjek, 2012). Boolean models can be converted into electronic circuits so that we can study the rich dynamics of Boolean networks using the signal processing theory (Xiao, 2009). Boolean models have been categorized into two main schema based on the timescales of biological events (Gershenson, 2004; Wang et al., 2012): synchronous (also called deterministic systems) and asynchronous schemes. In synchronous systems, all variables are assumed to have similar timescales and will be simultaneously updated, i.e., one unit will update all components simultaneously. In contrast, all variables will be updated non-simultaneously in asynchronous schemes if most of the timescales of biological events are different, i.e., each component will be updated at their own time unit (Wang et al., 2012).

Synchronous Boolean networks are based on the assumption that the state of a gene at a given time step is influenced by the state of a subset of genes in the network at the previous time step. The drawbacks of the synchronous systems are: they do not allow the temporal separation of changes in multiple regulatory events (Fauré et al., 2006), and they cannot measure differences in the speed of signal propagation as no two cells have the same properties. Hence, this results in differences in the rates of signal propagation between cells in the context of biological systems (Hwang and Lee, 2010).

The asynchronous networks only allow the update of one gene or component at a random time, resulting in a nondeterministic representation of the dynamics (Siebert, 2011). A drawback of the asynchronous scheme is that the resulting state transition graph is very complicated and encompasses many incompatible or unrealistic pathways (Fauré et al., 2006). There are also some modifications proposed for asynchronous Boolean models, such as non-deterministic asynchronous Boolean networks and deterministic asynchronous Boolean networks (Gershenson, 2004).

Models that combine synchronous and asynchronous transitions can illustrate the flexibility of the combination of different updating assumptions (Fauré et al., 2006). Berestovsky et al. (2013) proposed an integrated hybrid model (IHM) that combines Petri nets and Boolean networks to model integrated cellular networks. The hybrid model can be applied to three main cellular biochemical processes: signal transduction, transcription regulation and metabolism (Chaouiya, 2007; Berestovsky et al., 2013).

With the facilities of the existing Boolean modeling tools, Boolean networks have been successfully applied to yeast (Kauffman et al., 2003; Li et al., 2004; Davidich and Bornholdt, 2008; Kazemzadeh et al., 2012), flower morphogenesis of wall cress: Arabidopsis thaliana (Espinosa-Soto et al., 2004), Drosophila development (Sánchez and Thieffry, 2001; Albert and Othmer, 2003; Ghysen and Thomas, 2003; González et al., 2008; Sánchez et al., 2008; Fauré et al., 2014), haematopoiesis (Bonzanni et al., 2013), MOMP regulation (Tokar et al., 2013), the mammalian cell cycle or cell fate (Fauré et al., 2006; Schlatter et al., 2009; Grieco et al., 2013; Mombach et al., 2014; Ruz et al., 2014; Cohen et al., 2015), the light- and carbon-signaling pathways (Thum et al., 2003), apoptosis networks (Mai and Liu, 2009; Schlatter et al., 2009; Kazemzadeh et al., 2012; Schleich and Lavrik, 2013), the hepatocyte signal networks (Schlatter et al., 2012), NF-kappaB and IL-6 mediated by miRNA (Xue et al., 2013), and leukemia (Saez-Rodriguez et al., 2007; Wittmann et al., 2009; Hwang and Lee, 2010; Saadatpour et al., 2011, 2013; Zañudo and Albert, 2013; Campbell and Albert, 2014).

In their noteworthy analysis, Davidich and Bornholdt predicted the biological cell cycle sequence of fission yeast using a Boolean model, making 47 kinetic constants that were necessary for the ODE (ordinary differential equations) approach redundant, and it was assumed that the biochemical network was functioning in a parameter-insensitive way (Davidich and Bornholdt, 2008). Faure et al. extended the software GINsim and studied the dynamics of a Boolean model for the control of the mammalian cell cycle, with synchronous, asynchronous or hybrid treatment of concurrent transitions (Fauré et al., 2006). Moreover, the signal transduction network for abscisic acid has been proven to induce stomatal closure (Li et al., 2006).

Currently, the most proposed biological network inference methods to identify functional modules focus either on the definition of gene regulatory networks or, more recently, on the functional networks in which an edge indicates a functional relationship and a subset of genes that describe, explain or predict a biological process or phenotype (Lazzarini et al., 2016). Far less effort has been put into the consideration of constructing activation, inhibition, and protein decay networks that could indicate the direct roles of a gene (or its synthesized protein) as an activator or an inhibitor of a target gene. The major reason for this is that the hypotheses of the current Boolean models do not provide an intuitive way to identify the individual activation or inhibition pathways of the target gene. For example, a Boolean function determines the next state of the gene expression process as either *On* (activation) or *Off* (inhibition), taking into account the combined effects of the current state of its regulators (or the states of the associated regulators of the relevant gene expression processes) such as the Boolean function that describes the gene expression status of gene CycA (Hopfensitz et al., 2013; Ruz et al., 2014), which is given by:

(2) $$\begin{array}{r} \text{E2F }\&\;!\text{Rb }\&\;!\text{Cdc20 }\&\;!\text{(Cdh1 }\&\;\text{UbcH10})\;|\;\text{CycA }\&\;!\text{Rb }\&\\ \;!\text{Cdc20 }\&\;!\left(\text{Cdh1 }\&\;\text{UbcH10}\right)\to \text{CycA} \end{array}$$

where E2F, Rb, Cdc20, Cdh1or UbcH10 are potential genes that regulate gene CycA. As a result, a model of GRNs, including k number of genes, is constructed by the set of Boolean functions denoted as *F*, as given by Equation (3):

(3) $$\begin{array}{r} F=\left\{f_{i}|i=1,\ldots ,k\right\},\;f_{i}:\left\{0,1\right\}\to \left\{0,1\right\} \end{array}$$

The function given by Equation (2) for CycA combines both activation and inhibition pathways that require further inferences to determine the activation and inhibition parts. The roles of the gene activator and inhibitor in this example are not intuitively defined in the compressed Boolean function even though the compressed rule can be split into multiple subfunctions. A compressed Boolean function, which is defined as a rule that contains disjunctions with various subfunctions, can be divided into a set of *And* Boolean functions by the disjunction *Or*. For example, a Boolean function *P & Q* | *A & B & C &* (*D* |*E*) can be divided as follows:

$$\begin{array}{l} \forall =\left\{P\&Q,A\&B\&C\&D,\;A\&B\&C\&E\right\} \end{array}$$

where ∀ is a set of subfunctions. In a large GRN with many genes, this weakness becomes a significant problem in deciphering GRNs that are biologically meaningful. Furthermore, a single Boolean function determines the next status of a gene. However, this may not be true biologically because a gene may remain activated within a period of decay time when activator/activators are not present (Albert, 2004). Also, the original Boolean functions, as defined by Kauffman (Kauffman, 1969; Kauffman et al., 2003), are hard-wired with the assumption of biological determinism, but genetic regulations are fundamentally stochastic (Raj and van Oudenaarden, 2008; Xiao, 2009). The reason for this is that the expression of a gene usually encompasses the discrete and intrinsically random biochemical reactions involved in the processes of transcription and translation of mRNAs and proteins (Raj and van Oudenaarden, 2008). Probability Boolean networks (Shmulevich et al., 2002b; Shmulevich and Dougherty, 2005) are proposed to address the hard-wired issue by introducing stochastic components in which a gene is associated with multiple Boolean rules, and where each rule has a probability indicating the chance, and it will impact the target gene (Raj and van Oudenaarden, 2008). The total probability for all the rules of a gene is 1, and that means only 1 rule will be used to determine whether or not the target gene will be activated or inhibited at a particular time. Hence, the probability Boolean network model still inherits the major drawback of the conventional Boolean model in that a randomly selected rule ignores the fact that an unregulated gene can still be in the state of being activated. A gene can also be regulated competitively by another rule at the same time, such as the competitive inhibition (Fontes et al., 2000). Hence, the current Boolean models, in reality, may not be able to explain biological phenotypes accurately.

Therefore, to make the conventional Boolean functions clearer, we propose to focus on the general Boolean functions at the subfunction level taking into account the effectiveness of protein decay, and further split the subfunctions into the activation and inhibition domains. Because gene activation and inhibition are the two most fundamental components of complex cellular machinery, we use the term “fundamental Boolean functions” to denote these subfunctions. The biological meaning of the fundamental Boolean function is that it represents a regulatory function or regulatory complex function which can determine the activation or inhibition activity, respectively and directly. For example, Equation (2) is decomposed into six fundamental Boolean functions: CycA and E2F being TRUE trigger the activation function and Rb, Cdc20, (!E2F & !CycA) and (Cdh1 & UbcH10) being TRUE trigger the inactivation function.

In this paper, we propose a novel data-driven Boolean model, called the Fundamental Boolean Model (FBM), to draw insights into gene activation, inhibition and protein decay. This novel Boolean model provides an intuitive definition of the activation and inhibition pathways and includes mechanisms to handle protein decay as well as introducing uncertainty into Boolean functions. Furthermore, the new structure of the Boolean network allows us to propose a data mining method to extract the fundamental Boolean functions from genetic time series data. To prove the concept of the novel model, we implemented a platform using R language, called FBNNet, that is based on the proposed novel Boolean model, together with a novel data mining technique, to infer fundamental Boolean functions that visualize the dynamic trajectory of gene activation, inhibition and protein decay activities. The novel Boolean model is shown to infer GRNs with a high degree of accuracy using the time series data generated from an original cell cycle network.

The paper is prepared as follows: section Methods presents the proposed novel Boolean model and introduces a network inference method to infer networks for the model. In section Result and Discussion we present and discuss the details of the analysis results on a mammalian cell cycle network (Ruz et al., 2014), processed with the proposed FBM. Section Conclusions is the conclusions and discussion on the proposed FBM and experiment results.

## Methods

As discussed in the previous section, the hypotheses of the current Boolean models do not provide an intuitive way to identify the individual activation, inhibition, and protein decay of the target gene, as separate pathways. The gene activation and inhibition processes are the two primary fundamental processes of genetic regulation. Activation that increases the metabolism of drugs, for example, may result in significant drug regulatory effects, such as alterations in the metabolism of *in vivo* substances and vitamins as well as the activity of extrahepatic enzyme systems (Barry and Feely, 1990). Similarly, inhibition may result in significant clinical drug interactions that are produced by a wide range of drugs (Barry and Feely, 1990). Inhibition is usually divided into two groups: the first group consists of reversible inhibitors that can be easily reversed by dilution or dialysis because the interactions of this group are noncovalent with the various parts of the enzyme surface (Saboury, 2009); and the second group contains irreversible inhibitors that usually persist even during complete protein breakdown because their covalent bonds on the enzyme surface are strong (Saboury, 2009). Theoretically, under the assumption of an enzyme reaction exposed to the action of a reversible inhibitor, the degree of inhibition may be modeled as the reduction of the rate of reaction divided by the rate of uninhibited reaction (Saboury, 2009):

$$\begin{array}{l} i=\frac{V_{o}-V}{V_{o}} \end{array}$$

where V and *V*_*o*_ are the rates of inhibited and uninhibited reactions, respectively (Saboury, 2009). The degree of inhibition (*i*) introduces uncertainty into the target gene. Because enzyme activation also contains the concept of the reversible type of activators, we can redefine the degree of inhibition to the degree of enzyme reaction where V and *V*_*o*_ are the rates of affected and unaffected reactions, respectively. Therefore, we can convert the equation to a conditional probability measure, which is the probability of an event that occurs given another event has happened, to represent the propensity of an enzyme reaction to reduce or raise the enzyme-catalyzed reaction rate to the target gene. If a conditional probability value of an inhibitor is 1, the inhibitor is irreversible, and if the probability is <1 and more than 0, it is reversible.

In the conventional Boolean models Equation (1) represents the processes of gene activation and inhibition which do not consider the different behaviors of enzyme reactions such as reversible and irreversible reactions. Furthermore, the disappearance of an activator does not mean the emergence of an inhibitor, i.e., a Boolean activation function with a negation sign does not mean it has to be an inhibitor. Hence, there are justifiable reasons to separate the general Boolean function into the domains of activation and inhibition. To analyse the gene activation and inhibition networks, we abstracted the characteristics of enzyme activation and inhibition, such as mutual offsetting, reversible inhibition and the long-run degradation of a specific gene product (protein). Henceforth, we propose a novel Boolean model to construct dynamic activation and inhibition networks based on this abstraction. The following section explains the proposed novel model in detail.

### Definition of the fundamental Boolean model

Following the same pattern as the original definition of a Boolean model, we define our novel Boolean network as a graph G (X, *E*_*a*_, *E*_*d*_), where the node collection, *V* = {*v*_1_, *v*_2_, …, *v*_*n*_} corresponded to a group of states, *X* = {*x*_*i*_|*i* = 1, …, *n*} of size *n*, where each variable is only in one of two states: *On* (1) or *Off* (0), and the general edge set, *E*, was divided into two set of fundamental Boolean functions, *E*_*a*_ *and E*_*d*_, which are categorized by their regulatory functionalities, i.e., the activation and inhibition, rather than a single function, as in all conventional Boolean models. We denote this graph as a Fundamental Boolean Network (FBN) and the two sets of fundamental Boolean functions are defined as -

Fundamental Boolean functions of activation:

(4.a) $$\begin{array}{r} F_{a}^{i}=\left\{f_{a_{j}}^{i}|j=1,\ldots ,l_{a}\left(i\right)\right\},\;f_{a_{j}}^{i}:\left\{0,\;1\right\}\to \left\{-,\;1\right\} \end{array}$$

Fundamental Boolean functions of inhibition:

(4.b) $$\begin{array}{r} F_{d}^{i}=\left\{f_{d_{k}}^{i}|k=1,\ldots ,l_{d}\left(i\right)\right\},\;f_{d_{k}}^{i}:\left\{0,\;1\right\}\to \left\{-,\;0\right\} \end{array}$$

where $F_{a}^{i}$ and $F_{d}^{i}$ present a set of fundamental Boolean activiation and inhibition functions for the target gene *i*, respectively. “–” means the output of the function has no impact on the target gene. *l*_*a*_(*i*) denotes the total number of fundamental Boolean functions activating the target gene and *l*_*d*_(*i*) denotes the total number of fundamental Boolean functions deactivating the target gene. The output of a fundamental Boolean activation function is *TRUE* means the target gene will be activated and *FALSE* means the activation function has no impact on the target gene. Similarly, The output of a fundamental Boolean inhibition function is *TRUE* means the target gene will be inhibited and *FALSE* means the inhibition function has no impact on the target gene.

The proposed fundamental Boolean functions encapsulate the following biologically meaningful key ideas:

- A fundamental Boolean function is a simple transition rule that takes a minimum group of essential gene states as an input and determines the regulation effect on the target gene in the form of a Boolean value.
- In general, a fundamental Boolean function is a function that cannot be divided any further. Hence, a fundamental Boolean function can be regarded as a delegation of stereochemical reactions, such as the activity of a transcription factor formed by the binding of a few essential proteins; a combination of transcription factors formed containing multiple transcription factors; a transcription factor complex formed by binding a transcription factor with other proteins; and, conditions that constrain gene activity.
- A general assumption of the proposed fundamental Boolean functions is that the production of the coded protein for each gene at each time step is either completely activated or inhibited. A further assumption is that gene regulation can be entirely or partially affected by the recipe defined by a fundamental Boolean function that states how proteins bind to their target genes.
- The gene regulation time is embedded into Boolean updating schema based on the treatment of time. Under the synchronous scheme, all states have a unique successor, i.e., all nodes are updated at the same time, and all gene regulation processes are assumed to have completed upon the next time step. This scheme is simple but induces well-known artifacts (Fauré et al., 2006). With the asynchronous scheme, the result may be closer to biological reality but could be more computationally challenging to evaluate because every transition is updated at a different time step.

The output of the proposed functions reflects only the potential effectiveness of gene regulation on the target gene. Therefore, we need to make sure how confident we are to trust the regulatory functions that can impact on the target gene. As discussed previously, the degree of enzyme reaction can be replaced by the conditional probability of the event that an enzyme reaction can affect the target gene. Hence, the concept of conditional probability is then applied to measure the general confidence of the proposed functions. The following formulae, denoted as confidence measures, calculated the conditional probability of each fundamental Boolean function separated by the activation and inhibition of genes.

Confidence measure of activation:

(5.a) $$\begin{array}{l} C_{a_{j}}^{i}\left\lfloor f_{a_{j}}^{i}\left(A_{i}^{j}\left(t\right)\right)\right\rfloor =p\left(\sigma_{i}^{t+1}=1|A_{i}^{j}\left(t\right)=1\right)\\ \;=\frac{p\left(A_{i}^{j}\left(t\right)=1\;\cap \;\sigma_{i}^{t+1}=1\right)}{p\left(A_{i}^{j}\left(t\right)=1\right)} \end{array}$$

Confidence measure of inhibition:

(5.b) $$\begin{array}{l} C_{d_{k}}^{i}\left\lfloor f_{d_{k}}^{i}\left(D_{i}^{k}\left(t\right)\right)\right\rfloor =p\left(\sigma_{i}^{t+1}=0|D_{i}^{k}\left(t\right)=1\right)\\ \;=\frac{p\left(D_{i}^{k}\left(t\right)=1\;\cap \;\sigma_{i}^{t+1}=0\right)}{p\left(D_{i}^{k}\left(t\right)=1\right)} \end{array}$$

Where $\sigma_{i}^{t}$ represents the Boolean state of gene *i* at time t, and $\sigma_{i}^{t+1}$ represents the Boolean state of gene *i* at time t+1. ∩ is a logical *And* connector. $C_{a_{j}}^{i}\;and\;C_{d_{k}}^{i}$delegate the confidence function with the input of the fundamental Boolean functions $f_{a_{j}}^{i}$ and $f_{d_{k}}^{i}$, respectively. $A_{i}^{j}$ and $D_{i}^{k}$ represent the set of required inputs for the gene state functions, $f_{a_{j}}^{i}$ and $f_{d_{k}}^{i}$, respectively. $A_{i}^{j}\left(t\right)=1$ or $D_{i}^{k}\left(t\right)=1$ mean the required gene input of $f_{a_{j}}^{i}$ or $f_{d_{k}}^{i}$ at time *t* is satisfied. If the required gene input of a function is not satisfied, the conditional probability of the function is 0. The output of the confidence function is a probability indicating by what percentage we can believe the proposed functions have as the final impact on their target genes, when all conditions of their required input gene state are satisfied. The confidence measures of activation and inhibition introduce stochastic processes into gene regulation and they can be used to simulate stochastic epigenetic switches in nature. For example, chromosomal rearrangements can cause genes to show stochastic regulation such as position effect variegation in *Drosophila* and telomere position effect in yeasts (Edwards and Bestor, 2007). Another example is that the competition between the OxyR repressor, which is a regulator of antioxidant genes, and Dam (DNA adenine methyltransferase), which controls the activity of the *agn43* promoter, causes neither OxyR or Dam 100% efficient (Edwards and Bestor, 2007). We can simulate these examples by increasing or decreasing the confidence value, similar to the classic example of the *lac* operon discussed in (Edwards and Bestor, 2007).

There are debates on the decay time of mRNA/proteins in Boolean models that allow the gene to remain in the *On* state when no activators or inhibitors are present. Réka Albert proposed that this decay may happen in two time steps because the decay time of a protein in an inactive state is usually longer than the time taken for its synthesis (Albert, 2004). To encapsulate the factor of protein decay, we denote a function *f*_*decay*_ (given below) to summarize the requirements of protein degradation with input from the target gene *i* at time *t*:

(6) $$\begin{array}{r} f_{decay}\left(\sigma_{i}^{t},\vartheta \right)=\neg \left(\tau \leq \vartheta \right)\times \sigma_{i}^{t} \end{array}$$

where τ represents an incremental variable presenting the number of time steps that have been processed. The τ will be reset to 0 whenever there is any fundamental Boolean function having an effect on the target gene *i* at time *t* + 1. ϑ delegates the decay time period to reflect the fact that the attenuation or enhancement of the expression of mRNA requires time. ¬ represents a negation operator that changes a Boolean function from *TRUE* to *FALSE* or vice versa. × is a logical *And* operator. The output of the decay function *f*_*decay*_ is a Boolean value of *On* (1) at time *t* +1 if the gene state of σ_*i*_ at time *t* is *On* (1) within the tolerated time period or *Off* (0) at time *t* +1 when the tolerated time period is expired regardless of the gene state of σ_*i*_ at time *t*. We assume that the tolerated time period for protein decay has only one time step for short time series data. Short time series data contain an enormous number of genes but only a few observations; hence, knowledge of the mechanistic details and kinetic parameters cannot be extracted consistently from the data (Ernst et al., 2005; Wang et al., 2008; Chaiboonchoe, 2010). About 80% of published experimental data are short time series because the expenses involved in acquiring genetic data are not economic and the time taken to examine the patients is usually too short due to health issues (Ernst et al., 2005; Wang et al., 2008; Chaiboonchoe, 2010). With regard to long time series data, which contain more observations than short time series data, we assume the tolerated time period is in two time steps to match the decay assumptions of Réka Albert (Albert, 2004).

By combining Equations (4.a, 4.b), (5.a, 5.b), and (6) we propose the novel Boolean model (FBM) as:

(7) $$\begin{array}{l} \sigma_{i}^{t+1}=\left(f_{decay}(\sigma_{i}^{t},\vartheta )+\vee_{j\;=\;1}^{l_{a}(i)}\left\{P〚C_{a_{j}}^{i}\left\lfloor f_{a_{j}}^{i}(A_{i}^{j}(t))\right\rfloor 〛\right\}\right)\\ \;\times \neg \vee_{k\;=\;1}^{l_{d}(i)}\left\{P〚C_{d_{k}}^{i}\left\lfloor f_{d_{k}}^{i}(D_{i}^{k}(t))\right\rfloor 〛\right\} \end{array}$$

The role of $f_{decay}\left(\sigma_{i}^{t},\vartheta \right)$ in Equation (6) is to ensure that if no activators are present, the gene state σ_*i*_ at time *t* + 1 depends on the state of itself at time *t* if it is tolerated by the parameter ϑ, a decay time period. *P*〚*x*〛 is a Boolean function that takes a uniform distributed random number, μ, and an output of 1 if μ < x and 0 otherwise. V{*x*} denotes the logical connective function of *Or*, i.e., $V_{j=1}^{l_{a}\left(i\right)}${$F_{a}^{i}$ } = $f_{a_{1}}^{i}+\;f_{a_{2}}^{i}+\ldots +\;f_{a_{l_{a}\left(i\right)}}^{i}$ if all activation rules of gene *i* have the confidence measure value of 1. +is a logical *Or* operator. The output of the proposed model is the activation or inhibition status of the target gene *i* at time *t* +1. A general assumption of the proposed model is that all gene regulations are controlled by the proposed fundamental Boolean functions in the activation, inhibition and protein decay domains.

**Figure 2:**
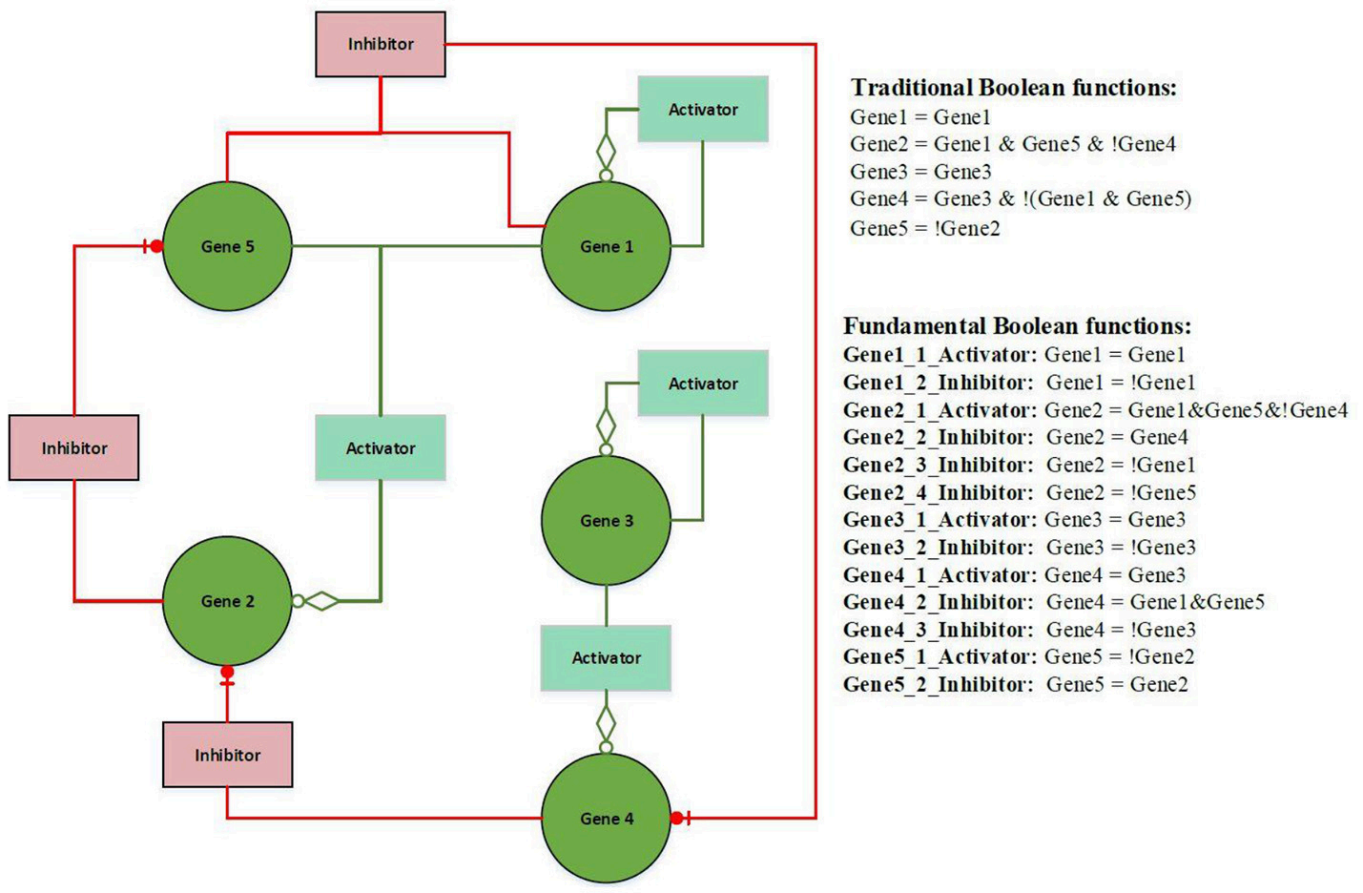
Example of a Fundamental Boolean Network. The icon box is denoted as a fundamental Boolean function. The red box is denoted as an inhibition function, and the light green box is denoted as an activation function. The green circle icon is denoted as a gene or a variable.

Figure 2 illustrates an example of FBN. The terms activator and activation function, and inhibitor and inhibition function are interchangeable throughout this paper. The left hand side presents a wiring diagram of FBNs; the top right hand side is a list of Boolean functions in the form of traditional Boolean models; the bottom right hand side has a list of fundamental Boolean functions. Both sets of functions have identical functionalities but the second set displays the rules separated by the types of activators and inhibitors (see Supplementary Information for an example of the calculation of fundamental Boolean function).

**Figure 3:**
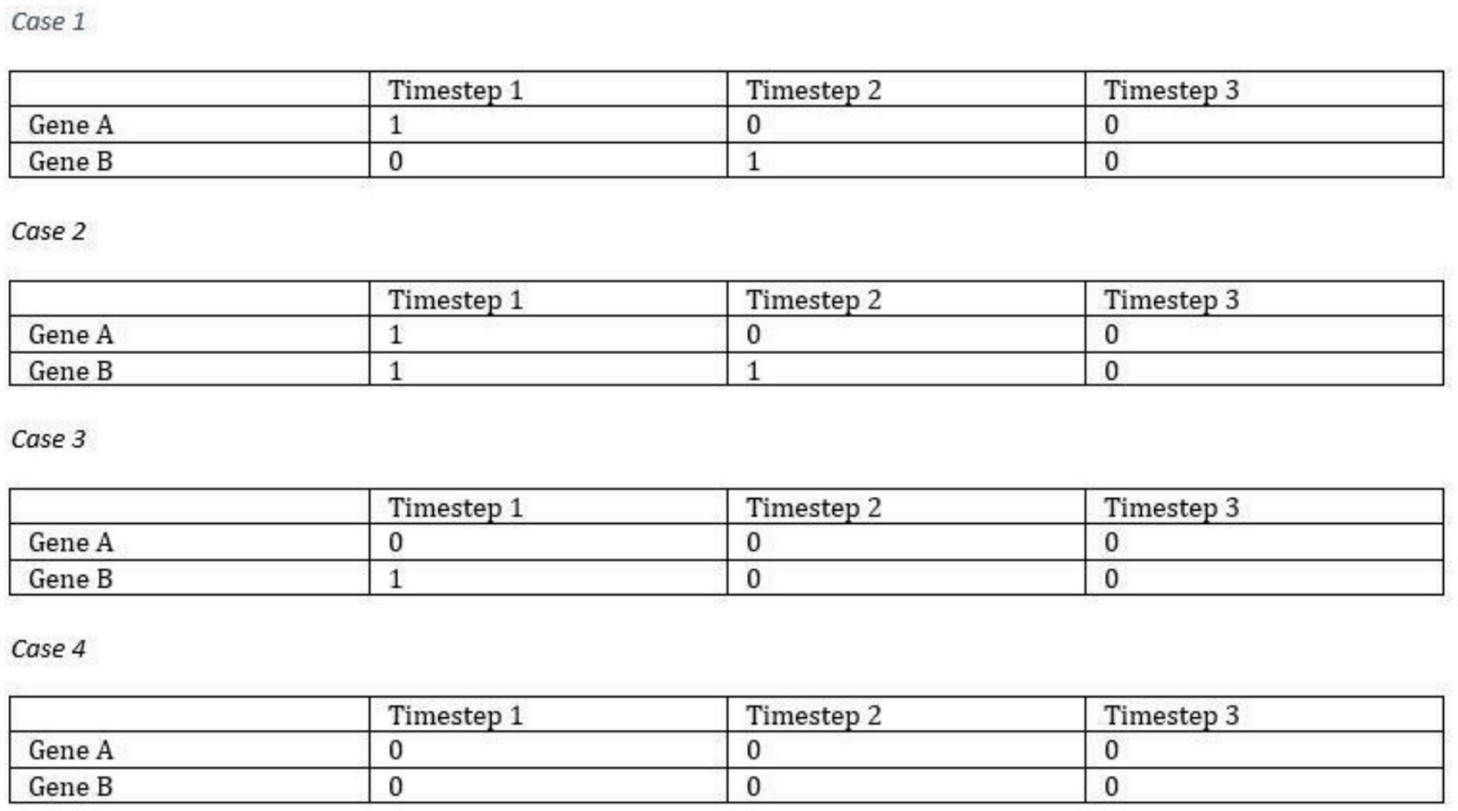
Simulation of the dynamic equilibrium of gene regulation.

The proposed model can simulate the dynamic equilibrium of gene regulation. Figure 3 illustrates an example of how the proposed model (FBM) can handle the dynamic equilibrium of gene regulation. In this example, gene A is an activator for gene B, but gene B is an inhibitor of gene A. The result is an equilibrium of both A and B. The FBM parameters are: the time step for protein decay is 1; the time step for a gene to complete its regulation process is 1; the confidence measure for each rule is 1 (100%). The Boolean updating schema is the synchronous scheme. In case 1, Gene B was activated by Gene A at the time step 2. Gene A was turned *Off* due to the protein decay at the time step 2. Gene B was turned *Off* at the time step 3, which is also due to the protein decay. In case 2, Gene A was inhibited by Gene B at the time step 2, and Gene B was continually boosted up by Gene A at the time step 2 but inhibited due to the protein decay at the time step 3. In case 3, Gene A was inhibited by Gene B and Gene B was turned *Off* due to the protein decay at the time step 2. In case 4, both genes were entrapped into a simple loop, namely, attractor due to the lack of activators to turn any of them *On*. All of the cases will be entrapped into the same attractor, i.e., the gene state of {0,0}.

The proposed Boolean model, i.e., Fundamental Boolean Model and the related Boolean network, i.e., Fundamental Boolean Network provides a novel mechanism to analyse the activation, inhibition and protein decay pathways intuitively. The potential application of the mechanism can be used to analyse the drug-related gene regulations because the inhibition pathway of a new drug can be revealed intuitively through the usage of the drug-related fundamental Boolean networks. The main challenge is how to extract the knowledge network (NK) from the drug-related dataset. The first knowledge network is normally referred to as the prior knowledge network (PNK) which encapsulates the biological knowledge already known for the main compounds involved in the process being studied (Traynard et al., 2017). Most of the PNKs came from the literature, and a very few of them came from the data mining related technologies. The PNKs retrieved from the literature were normally based on very small sample datasets: One reason is that the high expense involved in acquiring genetic data; another reason is that the period of patient's examination is usually either too short or fatal (Wang et al., 2008). Hence, we propose a new technology to extract the proposed fundamental Boolean networks from time series data. The following paragraphs illustrate the methodology to extract the fundamental Boolean model related networks.

### Inference network with an analytical cube

There are two main steps to inferring fundamental Boolean networks from time series data. The initial phase is to construct a cube type database to store all important precomputed measures. The precomputed measures are listed as follows (see SI for the example of each measure):

- Confidence Measures:
  Confidence measures are outputs of the confidence functions introduced in Equations (5.a) and (5.b) that indicate a conditional probability on the causality of regulation between a conditional gene, at time *t*, and the target gene, at time *t* + 1.
- Confidence Counter Measures:
  Confidence counter measures are similar to the confidence measures introduced in Equations (5.a) and (5.b) but are used to indicate the conditional probability on the causality of the target gene, at time *t*, which regulates the conditional gene at time *t* + 1. We denote the confidence counter measures as $C\forall_{a_{j}}^{i}$ and $C\forall_{d_{k}}^{i}$ for activation and inhibition, respectively, then we applied the following formula to calculate the value of the confidence counter. The outputs, $C\forall_{a_{j}}^{i}$ and $C\forall_{d_{k}}^{i}$ are conditional probabilities and, hence, the range of the value is between 0 and 1.
  Confidence counter measures of activation:
  (8.a) $$\begin{array}{l} C\forall_{a_{j}}^{i}\left\lfloor f_{a_{j}}^{i}\left(A_{i}^{j}\left(t+1\right)\right)\right\rfloor =p\left(A_{i}^{j}\left(t+1\right)=1|\sigma_{i}^{t}=1\right)\\ \;=\frac{p\left(A_{i}^{j}\left(t+1\right)=1\cap \;\sigma_{i}^{t}=1\right)}{p\left(\sigma_{i}^{t}=1\right)}\; \end{array}$$
  Confidence counter measures of inhibition:
  (8.b) $$\begin{array}{l} C\forall_{d_{k}}^{i}\left\lfloor f_{d_{k}}^{i}\left(D_{i}^{k}\left(t+1\right)\right)\right\rfloor =p\left(D_{i}^{k}\left(t+1\right)=1|\sigma_{i}^{t}=0\right)\\ \;=\frac{p\left(D_{i}^{k}\left(t+1\right)=1\cap \sigma_{i}^{t}=0\right)}{p\left(\sigma_{i}^{t}=0\right)}\; \end{array}$$
- Support Measures:
  Support measures are the percentage of transactions contain marched rules ($A_{i}^{j}\left(t\right)=1\cap \sigma_{i}^{t+1}=1$ and $D_{i}^{k}\left(t\right)=1\cap \sigma_{i}^{t+1}=0$) over all time steps in all samples. Let us denote the total number of time steps involved as ℵ
  $$\begin{array}{l} ℵ=\overset{s}{\underset{i=1}{\sum }}\left(t_{i}-1\right) \end{array}$$
  where *t*_*i*_ is the number of time steps of sample *i* and *s* is the total number of samples. The first time step of sample *i* is not included because the calculation of support measure involves two time steps (t and t + 1). We denote the support measures as $S_{a_{j}}^{i}$ and $S_{d_{k}}^{i}$ for activation and inhibition respectively. Therefore, the activation and inhibition of the support measurements for gene *i* are definded as:
  Support measure of activation:
  (9.a) $$\begin{array}{r} S_{a_{j}}^{i}=\frac{count\left(A_{i}^{j}\left(t\right)=1\cap \sigma_{i}^{t+1}=1\right)}{ℵ} \end{array}$$
  Support measure of inhibition:
  (9.b) $$\begin{array}{r} S_{d_{k}}^{i}=\frac{count\left(D_{i}^{k}\left(t\right)=1\;\cap \;\sigma_{i}^{t+1}=0\right)}{ℵ} \end{array}$$
- Conditional Causality Test:
  Some researchers claimed that causality is not a concept statistic and is not statistically ‘identifiable’ because a secluded causal hypothesis cannot be verified by using only observational data (Simcha et al., 2013). However, we believe that the direction between the conditional gene and the target gene is still able to be calculated based on the conditional probabilities between the conditional gene and the target gene using the following formulae:
  (10) $$\begin{array}{r} \text{Conditional causality test}=\frac{\text{Confidence measure}}{\text{Confidence counter measure}} \end{array}$$
  The formulae for calculating the confidence measures and the confidence counter measures have been discussed in Equations (5.a, 5.b) and (8.a, 8.b). The ratios and interpretations are based on plausibility reasoning theory: if gene *B* at time *t* causes gene *A*, at time *t* + 1, to be expressed, then the confidence of *p*(*A*_*t*+1_ = 1|*B*_*t*_ = 1) is equal to 1; in contrast, the reasoning that gene *B* at time *t* +1 is caused by gene *A* at time *t* may not be as strong as gene *A* at time *t* +1 caused by gene *B* at time *t* due to lack of information to support this reasoning. Hence, confidence *p*(*B*_*t*+1_ = 1|*A*_*t*_ = 1) is ≤ *p*(*A*_*t*+1_ = 1|*B*_*t*_ = 1). The ratio *p*(*A*_*t*+1_ = 1|*B*_*t*_ = 1) divided by *p*(*B*_*t*+1_ = 1|*A*_*t*_ = 1), can then be used as a test for the causality direction between genes *A* and *B*. We named this test a conditional causality test. This test can differentiate indirect regulators from direct ones because the indirect regulators will usually have weaker reasoning than the direct ones.
  Therefore, by giving confidence for a potential fundamental Boolean function and a confidence counter for the potential fundamental Boolean function, we can calculate the value of the conditional causality test and interpret it as follows:
  - If the value of the conditional causality test for target gene *A* and the conditional gene *B* is greater than 1, gene *A*, then, is regulated by gene *B*.
  - If the value is equal to 1, genes *A* and *B* are then regulated from each other.
  - If the value is lower than 1, there is no causal relationship, so the hypothesis that gene *B* regulates gene *A* can be rejected.
- Entropy and Mutual Information:
  The Shannon entropy theory provides a quantitative information measure about the probability of observing a particular symbol, or event, *P*_*i*_, within a given sequence
  $$\begin{array}{l} H=-\sum P_{i}logP_{i} \end{array}$$
  where *log* is a logarithm with base 2. The sequence is the sum of the probabilities of an event being either *On* or *Off* (Shannon, 1948; Shannon and Weaver, 1963; Liang et al., 1998). Moreover, mutual information is defined as:
  $$\begin{array}{l} M\left(X,Y\right)=H\left(Y\right)-H\left(Y|X\right)=H\left(X\right)-H\left(X|Y\right) \end{array}$$
  where *H*(*X*|*Y*) and *H*(*Y*|*X*) are the two conditional entropies that capture the relationship between sequences X and Y (Liang et al., 1998). *M*(*X, Y*) presents the remaining information between sequence X and the information shared between X and Y. The output state of X is determined by Y if *M*(*X, Y*) = H(X) (Liang et al., 1998). Hence, we can use this measure to find the causality relationship between genes.

The mutual information and Shannon entropy measures are assumption-free methods measuring unknown and complex associations; however, they have the limitation of overestimating the regulation relationships leading to possible failures, such as the inability to differentiate indirect regulators from direct ones, as cited by Liu et al. (Liu et al., 2016). To overcome this limitation, we combined the mutual information measures with the conditional causality test we proposed as an important mechanism to extract the regulatory rules. As mentioned previously, the conditional causality test can differentiate indirect regulators from direct ones and is complementary to the mutual information measures.

#### Orchard cube

**Figure 4:**
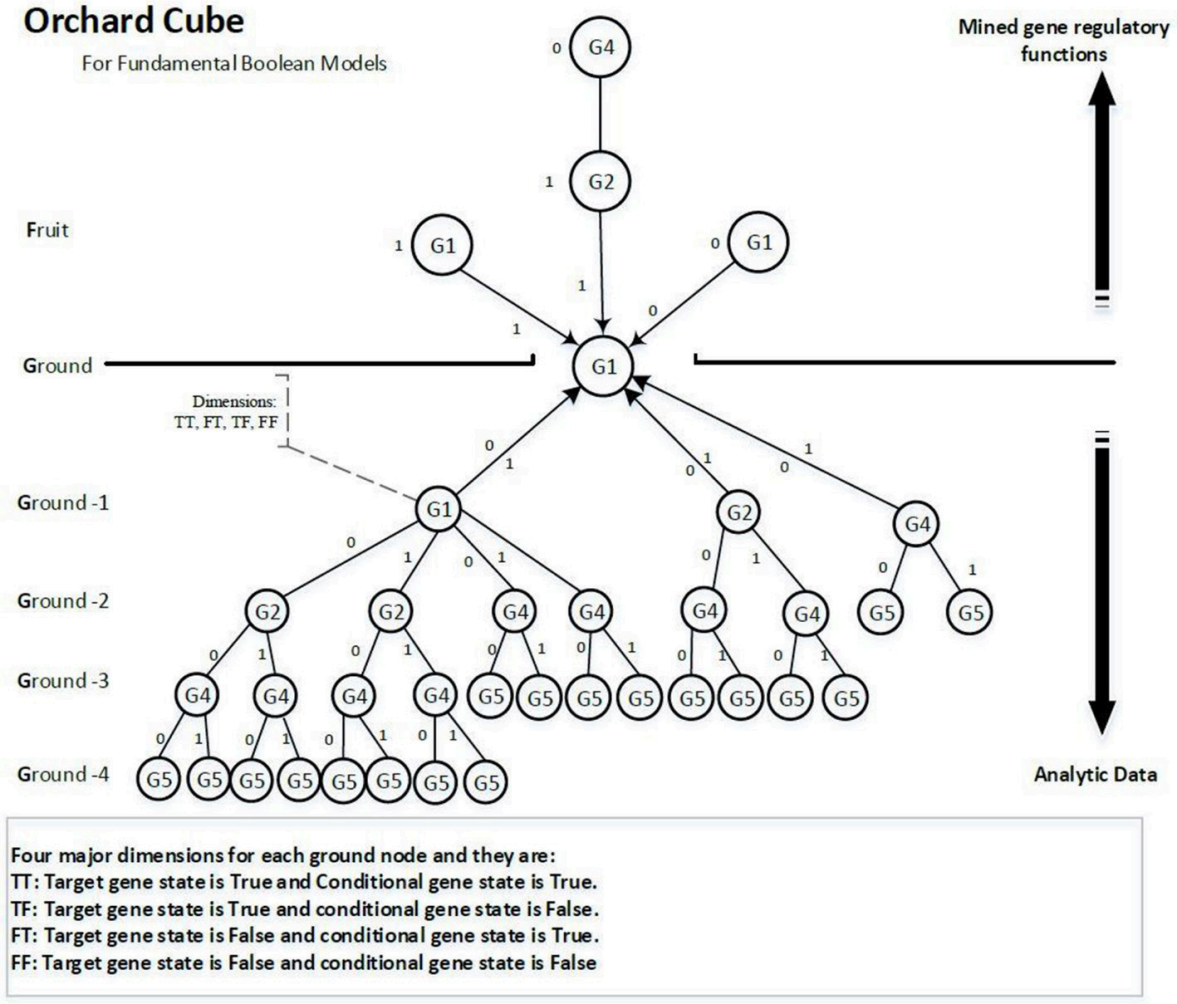
Illustration of an orchard cube.

A data cube is used to store precomputed measures for data mining. Many familiar genetic time series data are multidimensional, containing genes, time steps, and samples. Analysing multidimensional data could run into performance bottlenecks but precomputing a data cube can release performance bottlenecks by providing scalable mechanisms for fast access to the summarized data (Han et al., 2012). To infer the activators and inhibitors, we extend the data mining technique of bottom-up computation (BUC), which is an algorithm for the computation of sparse cubes from the Apex cuboid downward (Han et al., 2012) to a prefix tree type of cube, as shown in Figure 4. We call this cube the orchard cube because it looks like an orchard containing many fruit trees.

Each branch or link above ground on the tree is a regulatory function. Every node on a branch is a component of the regulatory function. Because regulatory functions are the knowledge we are looking for, we call them *fruit*. The gene nodes on the ground are called *seeds*. The training data are called *fertilizers* as they help the trees to grow larger (more confidence and; hence, more of certain functions). The reason we propose this type of cube is that we can distribute the computational costs to multiple computing threads or a cloud computing environment. The precomputing cube can be stored in any distributed database. The data training of every target gene is independent, which means we can distribute some target genes to a different computer to build their tree structures and then assemble all the distributed trees as an orchard forest. The network inference process is separated from the process of constructing cubes and has different pruning strategies. The separation between network inference and cube construction allows further development of scalable methods to extract genetic networks effectively and efficiently from comparatively fewer updates of the cube. Meanwhile, the cube can be consistently improved by integrating it with more time series data.

The nodes underground are analytical data that contain all possible regulatory functions for the target gene unless the NULL hypothesis rejects them. The nodes above ground comprise the extracted regulatory functions that have been mined from the nodes underground. Hence, if we define the collection of regulatory functions of a target gene as U^‘‘^ and the analytic data part as ˘U, then U^‘‘^ ∈ ˘U.

#### Pairwise dimensions

**Figure 5:**
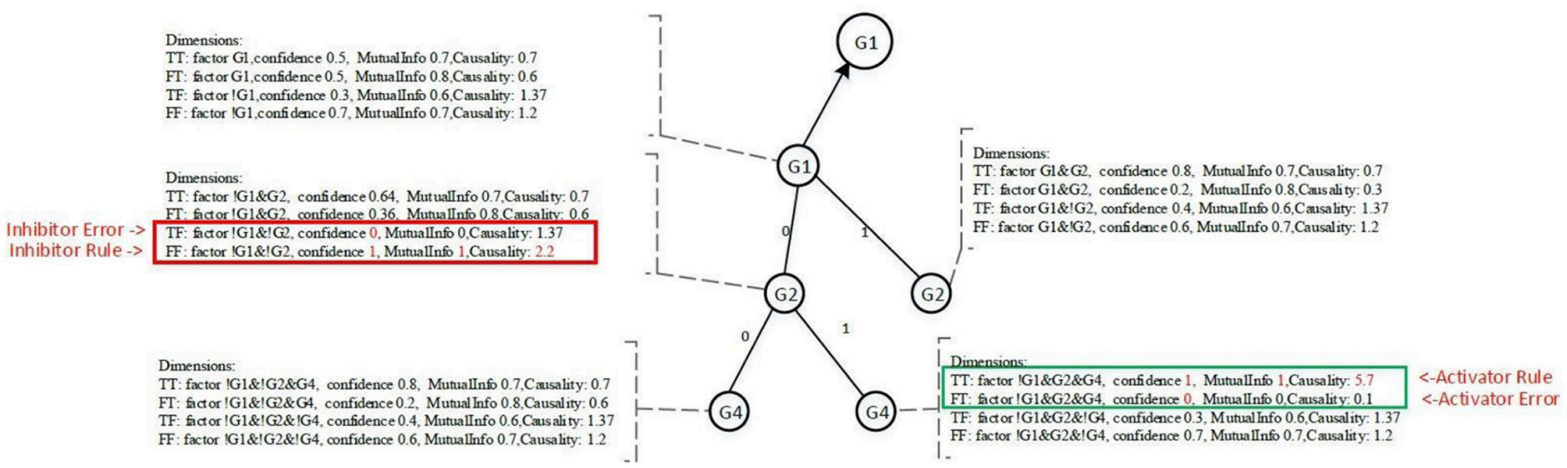
Sample nodes and measurement.

Each node contains four dimensions, i.e., four major groups of measures. Each dimension represents a potential regulatory function of the target gene. The four dimensions are denoted as TT, TF, FT, and FF, as shown in Figure 5, and are defined as follows:

- TT is when the target gene's state is TRUE, the current conditional gene state is TRUE, and all upstream conditional gene states are fixed;
- TF is when the target gene's state is TRUE, the current conditional gene state is FALSE, and all upstream conditional gene states are fixed;
- FT is when the target gene's state is FALSE, the current conditional gene state is TRUE, and all upstream conditional gene states are fixed;
- FF is when the target gene's state is FALSE, the current conditional gene state is FALSE, and all upstream conditional gene states are fixed;

All nodes under Ground-2 will have prefix gene states. For example, conditional gene, *G4*, of target gene *G1* at Ground-3 has its two upstream gene states fixed, i.e., *G1*(0) and *G2*(0), therefore, the four dimensions of the node *G4* are TT (*G1*|!*G1*&!*G2*&*G4*), TF (*G1*|!*G1*&!*G2*&!*G4*), FT (!*G1*|!*G1*&!*G2*&*G4*) and FF (!*G1*|!*G1*&!*G2*&!*G4*). Each dimension has a factor, also called a function statement, which represents the potential gene regulatory function. Dimensions TT and FT, FT and FF are two pairwise dimensions. The minimum confidence measures between TT and FT, FT and FF are error measures. The pairwise dimensions have the following characteristics:

P(TT)=1-P(FT) and P(TF)=1-P(FF)

For the pair dimensions, if we define one dimension to be the confidence measure that will have an impact on the target gene, the other dimension is then regarded as an error measure. This definition is equivalent to the definition of the essential Boolean state *x*_*i*_ that must match the requirements of

$$\begin{array}{lll} f\left(x_{1},\ldots x_{i-1},0,x_{i+1},\ldots ,x_{n}\right) & \neq & f\left(x_{1},\ldots x_{i-1},1,x_{i+1},\ldots ,x_{n}\right) \end{array}$$

for all *x*_1_, …*x*_*i*−1_, *x*_*i*_, *x*_*i*+1_, …, *x*_*n*_, where *f* is a Boolean function and *x*_*i*_ is a Boolean state (Fauré et al., 2006). The output of *f* is a Boolean value.

#### Orchard cube pruning

Constructing the cube requires building an optimal tree-type data structure from all possible combinations of all related genes up to a maximum depth. The computational cost grows exponentially. However, it is endurable because all precomputed genes will not go to next level to avoid redundant computations. For example, we have three genes, *A, B, C*, and the conditional probability of gene *A* and gene *B* regulating the expression of *C* is *p*(*C|A, B*). Because *p*(*C|A, B*) is equal to *p*(*C|B, A*), the branch for precomputing *p*(*C|B, A*) is not processed from the main tree. Hence, the computational cost of constructing the entire cube is affordable.

Before the initial pruning, we use Pearson's Chi-square test (Plackett, 1983) to test the NULL hypothesis that a target gene is independent of the gene selected as the conditional gene. All genes in GRNs apart from the target gene will be tested using this criterion to remove unrelated genes from Ground-2 when the *p*-value is over 0.05. This procedure reduces unnecessary root branches.

The Chi-square test only answers the question of whether or not a conditional gene can be associated with the target gene but fails to answer the question of whether or not it has a direct or indirect association because the principle of ‘guilt-by-association’ does not differentiate gene regulation from an indirect association (Childs et al., 2011). The only purpose for applying the Chi-square test is to find all potentially related genes. Because the number of associated genes with each target gene is lower than, or equal, to the total genes, the computational cost of processing each target gene can be reduced dramatically; however, this process is uneven.

#### The algorithm to construct the orchard cube

To build the orchard cube, we use the step-by-step algorithm below:

1. All genes are potential target genes; hence, we put all the genes as *seeds* on the ground level and partition them into *N* trees. *N* is the total number of genes. Because the construction of all trees is partitioned and parallel we explain the further steps for one tree only. The result is an orchard cube type of data structure that contains all precomputed measures, as discussed previously.
2. With target gene *i*, we first use the Chi-square test to test the NULL hypothesis of the target gene, against all other genes, for no relationship between them, as discussed previously.
3. We look through all potential regulatory genes and calculate all measures for the four dimensions, i.e., the TT, TF, FT, and FF, as discussed previously.
4. If the current underground level is lower than a value named *maxK* denoting the maximum underground level the tree can penetrate into, all potential regulatory genes, exclude the current gene and the genes that are already in the higher levels, will go to the next level.
5. In the next level, we repeat steps 3 and 4 until the current level is equal to *maxK* or all related genes are processed.
6. If the current level is equal to *maxK* or all relevant genes are processed, the construction of the current tree is then completed.
7. If all target genes have been processed, we then output the cube.

#### Inferences of fundamental Boolean networks

**Figure 6:**
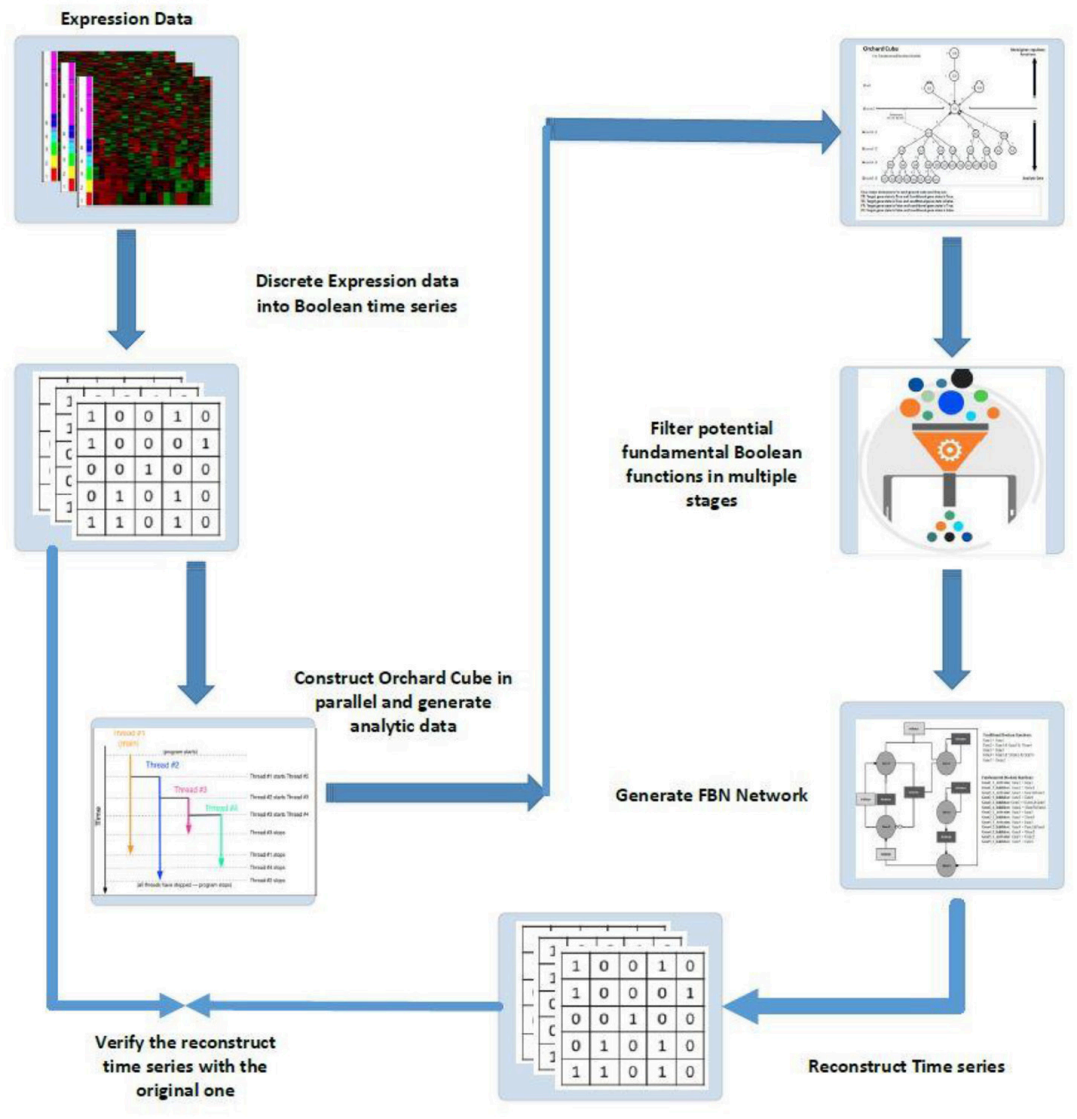
Schematic diagram of FBN modeling and network inferences: (1) discrete expression data into Boolean time series; (2) construct orchard cubes in parallel to generate analytical data and store all precomputed measures; (3) mine potential regulatory rules for all target genes through the constructed orchard cube based on some criteria; (4) generate the fundamental Boolean network; (5) use the generated network to reconstruct the input time series by giving the initial states of all original inputs and; (6) verify the reconstruction time series with the original series, if necessary, to gain confidence in the results.

The second phase is to mine fundamental Boolean functions from a cube type database structure. Figure 6 presents a schematic diagram of fundamental Boolean network inference.

As outlined in Figure 6, to infer FBNs we need to extract all essential measures, as explained previously, from a set of training data. The precomputed measures are then stored in a cube type database structure so the end users can generate different strategies to mine fundamental Boolean functions from the cube. The mined FBNs are then used to reconstruct time series to either trace the Boolean attractors or to verify the network generated by comparing the reconstructed time series data with the original training data.

Using the proposed orchard cube, we can infer interactions between the genes in the GRNs by filtering each tree's underground part based on the criteria listed below:

1. The conditional causality test value should be >1 or equal to 1.
2. The mutual information test should be equal to 1 or between a threshold and 1 when the time series data contain noise.
3. Discard the functions if they are matched with the following patterns because they are not essential states.
  $$\begin{array}{l} f_{a_{j}}^{i}\left(\text{A}\&\text{B}\&\text{C}\right)=f_{a_{j}}^{i}\left(\text{A}\&\text{B}\&!\text{C}\right)\text{ or}\\ f_{d_{j}}^{i}\left(\text{A}\&\text{B}\&\text{C}\right)=f_{d_{j}}^{i}\left(\text{A}\&\text{B}\&!\text{C}\right) \end{array}$$
4. The confidence measure value should be greater than a threshold, e.g., 0.7, to include some functions that contain noise. However, this criterion should be varied according to the noise level of the data.
5. Sort all functions remaining after step 4 by the mutual information measure, error measure, support measure and the number of genes input. Ideally, we want to keep the rules that have a more substantial support value with minimum errors.
6. The total number of fundamental Boolean functions for each type (activation or inhibition) of a gene is a limited to F_n_ based on different experimental requirements. However, in this study, we take F_n_ to be 5.

#### Reconstruction of time steps

The model, formulae and orchard cube we introduced in the previous section provide a complete mechanism to calculate the next gene state at time *t* + 1 by giving the gene state at time *t*. Therefore, we can reconstruct the time steps to any length by giving the initial gene state at time *t* = 0. The following list gives the primary usages of applying the model to reconstruct time steps:

Verify the reconstructed time series data against the original time series. The regulatory functions are a subset of the analytic data. If the functions are correct, we should be able to reconstruct time steps with the same initial states as the original time series. Because the asynchronous Boolean model produces an undetermined result, the reconstructed time series data might be different from the training time series data. Hence, we focus on demonstrating the FBM using synchronous Boolean schema only in this paper.

Reconstruct the hidden layers between the observed time steps. In short time series data, the gaps between observed time steps are very sparse. We can use the reconstructed time series to reveal the hidden layers by giving the initial Boolean state and keeping on generating the next time step based on the previous time step until the latest generated time step is identical to the next observed time step. By giving the two observed states *S*_*observe*_*d*__1__ and *S*_*observe*_*d*__2__, we reconstructed the time series data, as follows:

$$S_{reconstructed}=S_{1},S_{2}\ldots S_{k},\;S_{observed_{1}}=S_{1}\;and\;S_{observed_{2}}=S_{k}$$

The states between *S*_1_ and *S*_*k*_ are then denoted as missing time steps or hidden layers.

## Result and discussion

There are three main steps to verify the proposed logical model: (i) the specification of the updating schema of the model; (ii) the definition of the parameters of the model; (iii) the extraction of a regulatory network. As discussed in section Background, there is two main Boolean updating schema: synchronous and asynchronous based on the treatment of time. For the sake of simplicity, we apply the synchronous updating scheme to test the model. The parameters for the fundamental Boolean model are the protein decay, which, by default, is 1; the updating time step for each subfunction (fundamental Boolean function) is 1; the parameter of confidence of each subfunction is extracted using the methodology we proposed. To extract the fundamental Boolean network, we implemented an R package, namely *FBNNet* (Fundamental Boolean Network toolset, a prototype version is available at https://github.com/clsdavid/FBNNet_Lincoln), which can build an Orchard type of cube and mine FBNs from the cube. The *FBNNet* tool can find attractors under the two main Boolean updating schema and plot a static regulatory graph as well as a dynamic regulatory graph. The following paragraphs describe the main experiment we conducted.

### Experimental design and dataset

**Figure 7:**
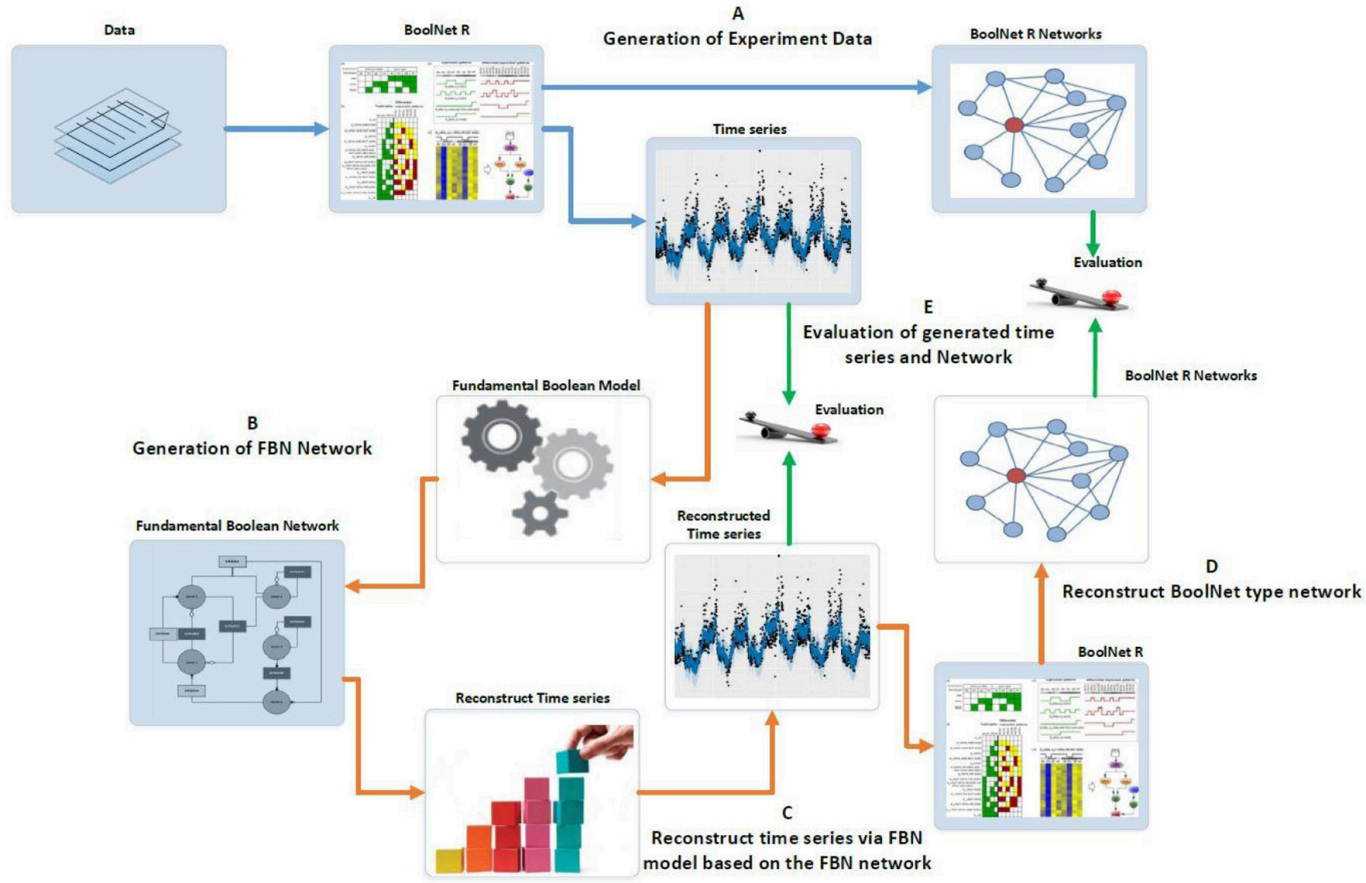
Experiment design of evaluating the Fundamental Boolean network inference. The blue arrows represent the processes using BoolNet and brown arrows represent the processes using our FBNNet tools. The green arrows represent the evaluation process. **(A)** We use the BoolNet script loadNetwork.R to load pre-defined networks from files and then generate the time series and networks. **(B)** We use the time series generated from BoolNet and the new R package, FBNNet, to generate FBNs. **(C)** We reconstruct the time series via the FBM. **(D)** To evaluate the FBM, we rebuild the BoolNet type network based on the reconstructed time series; and **(E)** we evaluate the FBN inference methods by comparing the generated time series and the generated BoolNet type of network with the original time series and network that were generated in step A.

The experiments conducted and described here intend to prove the concept of the new Boolean Model, i.e., the FBM. Figure 7 outlines the experiment design as a benchmark to compare the results generated via *BoolNet* (Müssel et al., 2010) with those consequently reconstructed from the new R package, *FBNNet*. The *BoolNet* package was demonstrated in the tutorial of (Hopfensitz et al., 2013) and the study of Ruz et al. (2014). The advantage of the *BoolNet* package over other existing tools, such as GINsim (Gonzalez et al., 2006), BooleanNet (Albert et al., 2008) and BN/PBN toolbox in Matlab, is the support of all three network types. *FBNNet* is a new R package that we have implemented for testing the concept of the FBM, and it has been fully integrated with the algorithms and concepts we introduced in section Methods.

**Figure 8:**
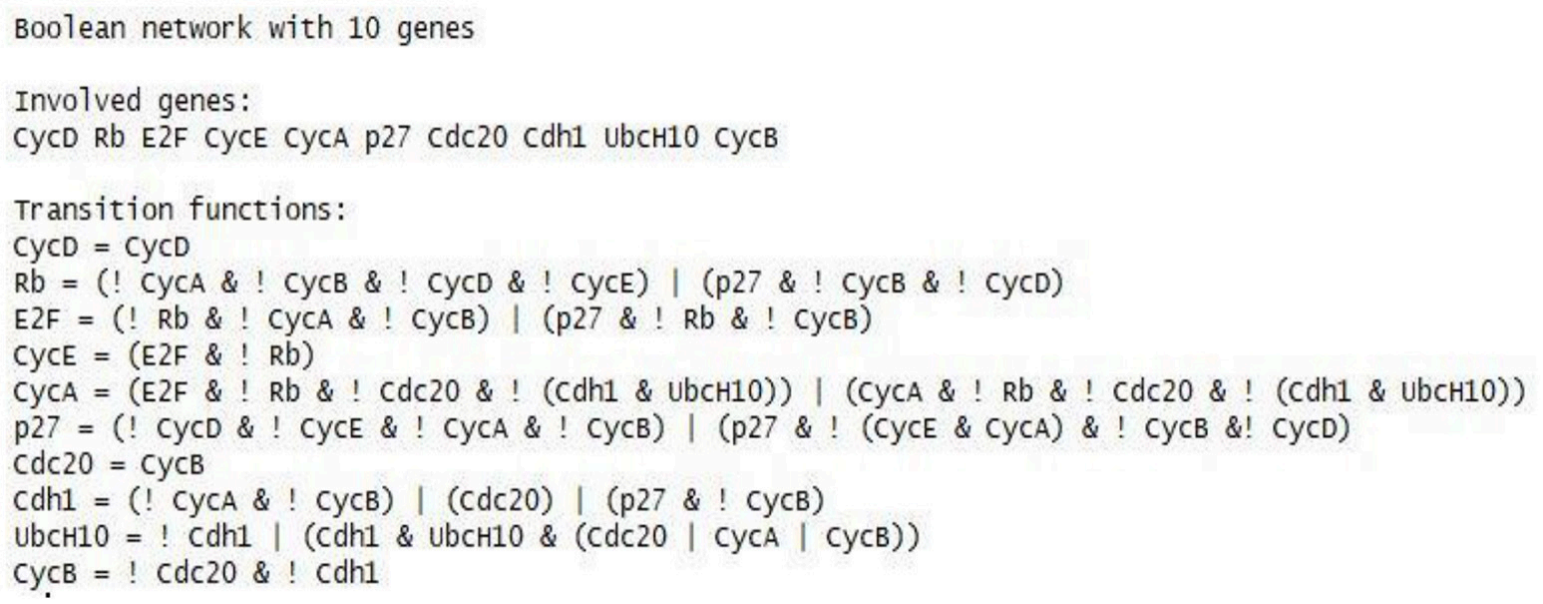
Known mammalian cell cycle networks provided by *BoolNet*.

Many new algorithms can be verified using the simulated datasets derived from several known regulatory networks, and the results can be compared with other known regulatory networks. In this paper, we propose to use the mammalian cell cycle network listed in Figure 8, to generate test data. The mammalian cell cycle networks have been well demonstrated in (Hopfensitz et al., 2013). Naturally, The regulation of the mammalian cell cycle leads to the reproduction of the genome of a cell either in Synthesis or S phase and its division involves two daughter cells (Mitosis, or M phase); and the M phase itself contains four different sub-phases (prophase, metaphase, anaphase, and telophase) (Fauré et al., 2006). The S and M phases involve two gap phases, namely G1 and G2 (Fauré et al., 2006).

To generate the experimental data, firstly, we used the command *loadNetwork* from *BoolNet* to load the cell cycle network specified in the text files: cellcycle.txt, as shown in Figure 8. Secondly, we use the method *generateTimeSeries* of *BoolNet* to generate 1024 noiseless sample data with 43 time steps for the cell cycle network, with all default settings, i.e., the parameter *type* is synchronous, the parameter *noiseLevel* is 0, and the parameter perturbations is 0. Each sample contains the same 10 mammalian cell cycle genes, i.e., CycD, Rb, E2F, CycE, CycA, p27, Cdc20, Cdh1, UbcH10, and CycB, as the study conducted by Hopfensitz et al.(2013). The generated 1024 sample data are the dataset used for this experiment. Each sample contains 43 time steps in sequence. All the initial states of the 1024 samples are unique containing the complete combination of 2^10^ changes. Hence, the number of genes that are expressed in each sample is variant.

Because the proposed Boolean rule definition is very different from the traditional Boolean rules, i.e., intuitive rules vs compressed rules (not intuitive) as discussed in section Background, we cannot compare it with other networks generated by other tools directly as it would not be a fair comparison. Hence, the best way to evaluate the generated FBNs is to use them to reconstruct time series data with the same initial states and then compare with the training time series data. The reason is that under the synchronous model if the generated network is correct, the network should be able to produce the same time series data as the training time series data with the same initial states.

The evaluation matrix for the time series comparison we have adopted is ER (Error rate), AR (Accurate rate), MMR (Mismatched rate) and PMR (Perfect matched rate). The matrix is defined as follows:

$$\begin{array}{l} ER=\frac{\sum_{i=1}^{n}\text{num of unmatched state per sample(i)}}{n}\\ AR=\frac{\sum_{i=1}^{n}\text{num of matched state per sample(i)}}{n}=1-ER\\ PMR=\frac{\text{Num of 100\% matched sample matrixes}}{n}\\ MMR=\frac{\text{Num of unmatched sample matrixes}}{n}=1-PMR \end{array}$$

where n is the total number of samples. Time series data here are referred to a list of sample matrixes, and each sample matrix contains gene states (Boolean value). Hence, a sample data means a sample matrix in this paper.

### FBN of cell cycle and its validation

The FBN for the sample genes and the FBN for the cell cycle genes were inferred via the R package *FBNNet*, as shown in Table 1.

**Table 1:** Inferred FBN cell cycle network.

**Fundamental Boolean Network with 10 genes Genes involved:**
CycD, Rb, E2F, CycE, CycA, p27, Cdc20, Cdh1, UbcH10, CycB
**Multiple Transition Functions for CycD with decay value = 1:**
CycD_1_Activator: CycD = CycD (Confidence: 1, TimeStep: 1)
CycD_2_Inhibitor: CycD = !CycD (Confidence: 1, TimeStep: 1)
**Multiple Transition Functions for Rb with decay value = 1:**
Rb_1_Activator: Rb = !CycD&p27&!CycB (Confidence: 1, TimeStep: 1)
Rb_2_Activator: Rb = !CycD&!CycE&!CycB&!CycA (Confidence: 1, TimeStep: 1)
Rb_3_Inhibitor: Rb = CycD (Confidence: 1, TimeStep: 1)
Rb_4_Inhibitor: Rb = CycB (Confidence: 1, TimeStep: 1)
Rb_5_Inhibitor: Rb = CycA&!p27 (Confidence: 1, TimeStep: 1)
Rb_6_Inhibitor: Rb = CycE&!p27 (Confidence: 1, TimeStep: 1)
**Multiple Transition Functions for E2F with decay value = 1:**
E2F_1_Activator: E2F = !Rb&!CycA&!CycB (Confidence: 1, TimeStep: 1)
E2F_2_Activator: E2F = !Rb&p27&!CycB (Confidence: 1, TimeStep: 1)
E2F_3_Inhibitor: E2F = Rb (Confidence: 1, TimeStep: 1)
E2F_4_Inhibitor: E2F = CycB (Confidence: 1, TimeStep: 1)
E2F_5_Inhibitor: E2F = CycA&!p27 (Confidence: 1, TimeStep: 1)
**Multiple Transition Functions for CycE with decay value = 1:**
CycE_1_Activator: CycE = !Rb&E2F (Confidence: 1, TimeStep: 1)
CycE_2_Inhibitor: CycE = !E2F (Confidence: 1, TimeStep: 1)
CycE_3_Inhibitor: CycE = Rb (Confidence: 1, TimeStep: 1)
**Multiple Transition Functions for CycA with decay value = 1:**
CycA_1_Activator: CycA = !Rb&E2F&!Cdc20&!UbcH10 (Confidence: 1, TimeStep: 1)
CycA_2_Activator: CycA = !Rb&CycA&!Cdc20&!UbcH10 (Confidence: 1, TimeStep: 1)
CycA_3_Activator: CycA = !Rb&CycA&!Cdc20&!Cdh1 (Confidence: 1, TimeStep: 1)
CycA_4_Activator: CycA = !Rb&E2F&!Cdc20&!Cdh1 (Confidence: 1, TimeStep: 1)
CycA_5_Inhibitor: CycA = Rb (Confidence: 1, TimeStep: 1)
CycA_6_Inhibitor: CycA = Cdc20 (Confidence: 1, TimeStep: 1)
CycA_7_Inhibitor: CycA = !E2F&!CycA (Confidence: 1, TimeStep: 1)
CycA_8_Inhibitor: CycA = Cdh1&UbcH10 (Confidence: 1, TimeStep: 1)
**Multiple Transition Functions for p27 with decay value = 1:**
p27_1_Activator: p27 = !CycD&!CycE&!CycB&!CycA (Confidence: 1, TimeStep: 1)
p27_2_Activator: p27 = !CycD&!CycA&!CycB&p27 (Confidence: 1, TimeStep: 1)
p27_3_Activator: p27 = !CycD&!CycE&!CycB&p27 (Confidence: 1, TimeStep: 1)
p27_4_Inhibitor: p27 = CycD (Confidence: 1, TimeStep: 1)
p27_5_Inhibitor: p27 = CycB (Confidence: 1, TimeStep: 1)
p27_6_Inhibitor: p27 = CycA&!p27 (Confidence: 1, TimeStep: 1)
p27_7_Inhibitor: p27 = CycE&!p27 (Confidence: 1, TimeStep: 1)
p27_8_Inhibitor: p27 = CycE&CycA (Confidence: 1, TimeStep: 1)
**Multiple Transition Functions for Cdc20 with decay value = 1:**
Cdc20_1_Activator: Cdc20 = CycB (Confidence: 1, TimeStep: 1)
Cdc20_2_Inhibitor: Cdc20 = !CycB (Confidence: 1, TimeStep: 1)
**Multiple Transition Functions for Cdh1 with decay value = 1:**
Cdh1_1_Activator: Cdh1 = Cdc20 (Confidence: 1, TimeStep: 1)
Cdh1_2_Activator: Cdh1 = !CycA&!CycB (Confidence: 1, TimeStep: 1)
Cdh1_3_Activator: Cdh1 = p27&!CycB (Confidence: 1, TimeStep: 1)
Cdh1_4_Inhibitor: Cdh1 = !Cdc20&CycB (Confidence: 1, TimeStep: 1)
**Multiple Transition Functions for UbcH10 with decay value = 1:**
UbcH10_1_Activator: UbcH10 = !Cdh1 (Confidence: 1, TimeStep: 1)
UbcH10_2_Activator: UbcH10 = Cdc20&UbcH10 (Confidence: 1, TimeStep: 1)
UbcH10_3_Activator: UbcH10 = UbcH10&CycB (Confidence: 1, TimeStep: 1)
UbcH10_4_Activator: UbcH10 = CycA&UbcH10 (Confidence: 1, TimeStep: 1)
UbcH10_5_Inhibitor: UbcH10 = Cdh1&!UbcH10 (Confidence: 1, TimeStep: 1)
**Multiple Transition Functions for CycB with decay value = 1:**
CycB_1_Activator: CycB = !Cdc20&!Cdh1 (Confidence: 1, TimeStep: 1)
CycB_2_Inhibitor: CycB = Cdh1 (Confidence: 1, TimeStep: 1)
CycB_3_Inhibitor: CycB = Cdc20 (Confidence: 1, TimeStep: 1)

As shown in Table 1, three primary parameters are bound with this novel FBN, and they are confidence (Equation 5), protein decay (Equation 6) and time step. The two parameters, time step and protein decay, were configured to 1 to match the way of generating the experimental data via the method generateTimeSeries of *BoolNet*. The method generateTimeSeries of *BoolNet* does not provide a configurable parameter for protein decay but has a default value of 1 (time step), embedded inside its logic.

A significant difference from other existing Boolean models is that the inferred FBN splits the Boolean functions of the mammalian cell cycle into the domain of activation and inhibition intuitively as shown in Table 1. Each gene could be regulated by multiple activation rules or inhibition rules at the same time. The result in Table 1 shows that the uncertainty of the process can be incorporated into the model. All FBM functions have the parameter confidence of 1, means the result is extremely accurate. To verify the result, we used the initial states from the training time series dataset to regenerate the same size data set using the novel concept of FBM (Equation 7) under the synchronous updating schema (same as the schema when the training dataset is generated). The regenerated dataset, then, is compared with the training time series dataset.

As shown in Table 2, all reconstructed time series data from the mammalian cell cycle network are identical to the pre-generated time series data with 100% in both AR and PMR. 100% of AR and PMR means all regenerated time series data are matched with the training time series dataset. Hence, we believe the inferred FBN cell cycle network with the proposed FBM is an alternative way to represent the mammalian cell cycle network but provides more information to draw insights into the activation and inhibition pathway of the mammalian cell cycle network.

**Table 2:** Experimental results for reconstructed time series data.

Network	**Number of samples**	**Number of time steps**	**ER**	**AR**	**PMR**	**MMR**
Cell cycle	1,024	43	0	100%	100%	0

**Figure 9:**
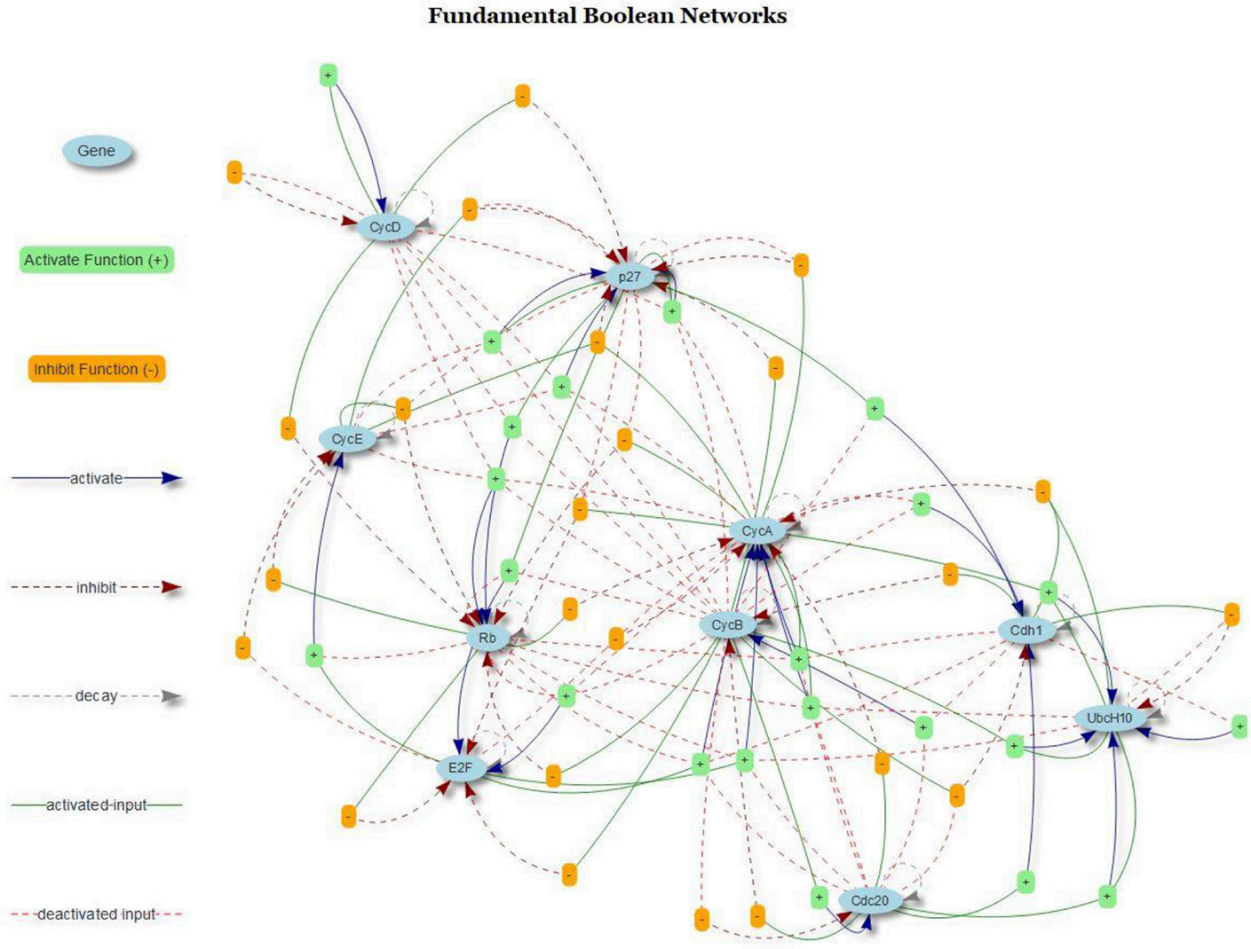
Cell Cycle FBN. The light blue elliptical icons represent genes; the orange box icons represent inhibition functions, and the light green box icons represent activation functions. The dark blue arrows represent activation, dark red arrows represent inhibition, and gray arrows represent protein decay.

Regarding FBNs, Figure 9 present the regulatory graph of the cell cycle network. The graph is generated by integrating the mined FBNs with the R package visNetwork (see online documentation http://datastorm-open.github.io/visNetwork/).

As shown in Figure 9, the internal relationships between genes are displayed. Hence, we can explore how these genes activated and inhibited other genes by tracing the input genes and the target genes via activators and inhibitors. The FBN of cell cycle contains three type of connections in the domains of gene activation, gene inhibition and protein decay.

### Attractors

**Figure 10:**
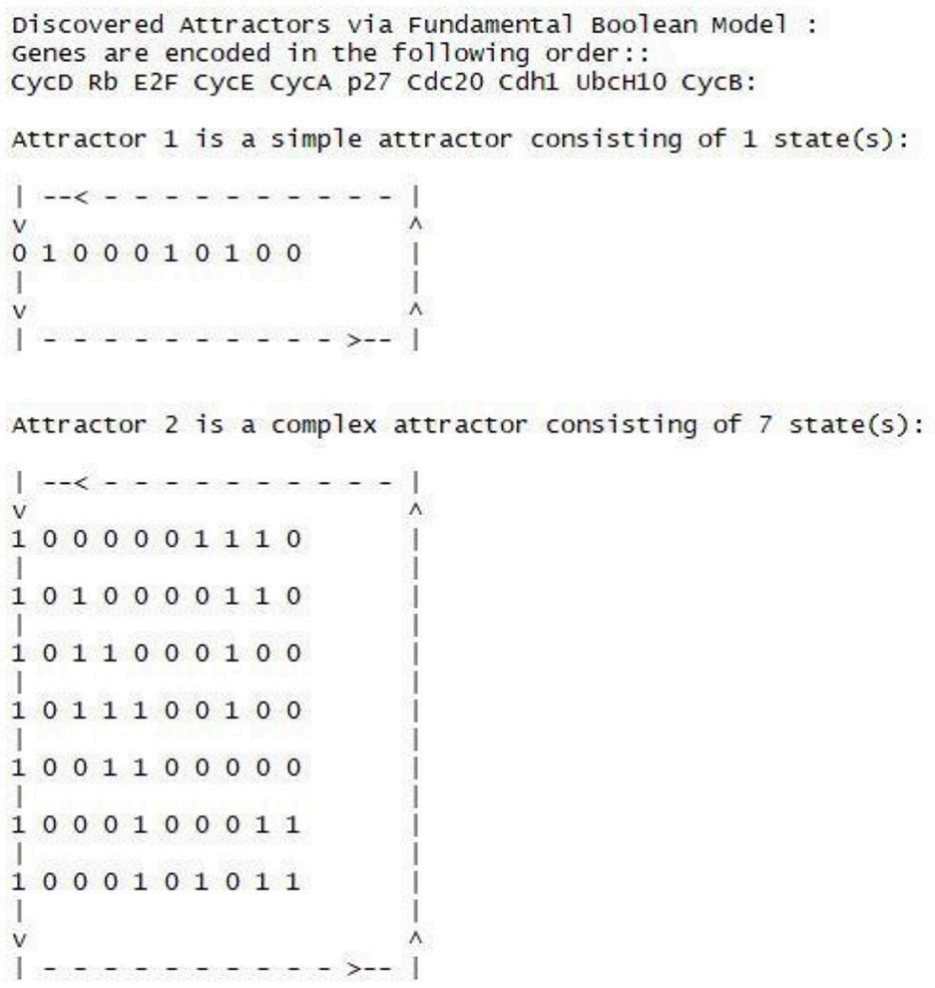
Synchronous attractors of the cell cycle fundamental Boolean model.

Attractors are defined as recurrent cycles of states (Hopfensitz et al., 2013), which are of particular interest in Boolean modeling. When a network reaches an attractor, it entraps a cycle most of the time until external perturbations happen to alter some essential genes' production to let the network get out of the entrapment. With the simplest synchronous assumption, we yield 2 attractors as shown in Figure 10. One is a stable attractor, and the other is cycle attractor. The attractors are equivalent to the findings that have been reported in (Fauré et al., 2006; Hopfensitz et al., 2013). Hence, the FBN of the cell cycle can produce the same attractors as other Boolean models did.

The first attractor is a simple attractor with only Rb, p27 and Cdh1 active, and is associated with the phase G0 or cell quiescence (Fauré et al., 2006). The CycD represents the whole cdk4/6-Cyclin D complex, and cdk4/6 is a cyclin-dependent kinase (cdk) partners.

**Figure 11:**
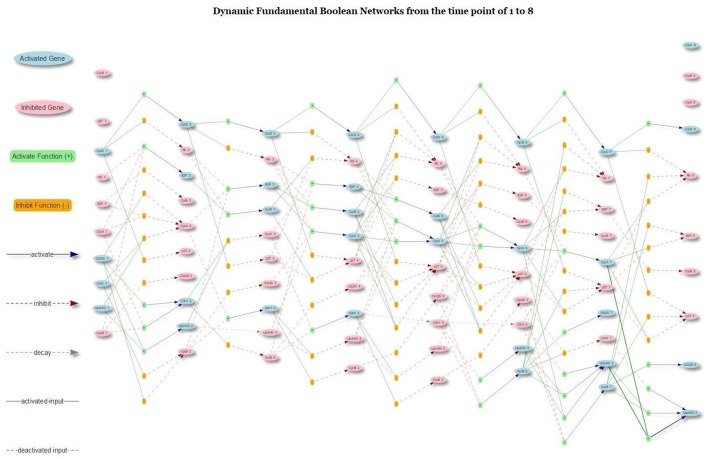
The dynamic trajectory of attractor 2. The underscore mark “_” indicates the time step that the gene was located.

The richness of the proposed model was its dynamic networks that provided a complete trajectory of gene activation, inhibition and protein decay. Figure 11 presents the dynamic trajectories of attractor 2, which explicitly display internal mechanism of gene regulation under the domains of gene activation, inhibition and protein decay. The presence of CycD lead to other seven stable dynamical cycles; each cycle is made of a sequence of seven successive states (attractor 2). The hidden pathways of the attractor 2 are not clear with any conventional Boolean model and related networks. However, with the proposed model and type of networks, the pathways of the attractor 2 are explicitly displayed. For example, in Figure 11, the CycD deactivates Rb and p27. The Rb, in turn, activates E2F, which is a family of dimeric transcription factors. As a consequence, the E2F activates CycE and CycA. The activation of CycE and CycA continually maintains the inhibition of Rb and p27. Moreover, Cdh1 is another critical element, which is an activator delegating the APC, an essential E3 ubiquitin ligase. The present Cdh1 dissociates the CycB directly and keeps inhibiting UbcH10. Rb phosphorylates E2F, CycA, and CycE, and the promotion of p27 enhances Cdh1. Rb continues to be activated without the

interruption of CycB, CycA, CycE, and CycD. The UbcH10 at the time step of 3 is inhibited as the consequence of protein decay (see the gray dash arrow line) because none of its activation and inhibition functions has an impact on it.

As shown through the demonstration of the proposed model with the mammalian cell cycle, we demonstrate that we could use the proposed orchard cube to infer the GRNs of the mammalian cell cycle. The outcome shows that if the network inferred was 100% correct, the reconstructed time series should match 100% with the original training dataset. However, this assumption was based on the degree of completeness of the initial training dataset when used with the synchronous Boolean schema.

The cell cycle FBN reveals the internal gene activation, inhibition and protein decay mechanism. Although the generated new Boolean cell cycle network is very different from any other Boolean network, it still can generate the same attractors as others such as in the study of Fauré et al. (2006), which means our proposed Boolean model is a new extension of the conventional Boolean models. Compared with the traditional Boolean cell cycle network as in the study of Hopfensitz et al. (2013), our network splits a complex rule into multiple rules under the domain of activation and inhibition and provides more insights into the dynamics of the pathways than those given by others. With FBN, a node is associated with multiple rules under two types (activation and inhibition type). The protein decay is also considered as a link of gene transition; and hence, the FBN contains three type of links: activation, inhibition and protein decay (as shown in Figure 11). This new feature can facilitate scientists to develop pharmaceutical agents by analyzing the related fundamental Boolean network and simulating the perturbation due to drugs.

We also show the dynamic trajectories for the attractor 2. The main advantage was that it illustrated the relationships in the domains of activation, inhibition, and protein decay to facilitate scientists in understanding the intrinsic genetic regulations. The downside was that the FBN might contain too many links. Comparing the rules shown in Table 1 and the original rules in Figure 8, our network might contain too many rules, but all of these rules are extracted from our data-driven model with an outstanding confidence value. Hence, the FBN of the cell cycle is fine-grained, and the original Boolean network as shown in Figure 8 is coarse-grained. Besides, we could limit the number of rules per type (activator and inhibitor). However, reducing the number of rules per type might reduce the correctness of the inferred network.

The current version of *FBNNet* was implemented as a prototype for the proposed FBM using pure R language without any performance optimization enhanced. Hence, to generate the experimental results, it requires approximately 200 s with parallel computing and 530 s without parallel computing. The machine we used to experiment is a laptop, which is made by Acer™, a model of Aspire V 17 Nitro. The R does not provide the facility of parallelisation directly, and we have to use other packages, namely, “parallel,” “foreach,” and “doParallel” to do the parallel computing. The performance of these packages are unknown, and they may not provide the real power of parallel computing as good as C or C++. However, they are good enough to prove the concept of the proposed novel Boolean model. *BoolNet* uses C to speed up its performance in constructing the cell cycle network, and hence, is faster than the current version of *FBNNet*. In contrast, our method is used to derive the intuitive activation and inhibition pathways and hence requires more computational times. In addition, the proposed orchard cube is used to store all precomputed measures for all potential fundamental Boolean functions in case we need to mine FBN from short time series data. Hence, it requires more time to process and keep as many potential rules as possible.

Finally, we proposed a way to reconstruct any missing time steps by estimating all fundamental Boolean functions' *TRUE* or *FALSE* values affecting their target genes by verifying the input states to be matched with the requirements of the functions. The time interval between time steps was a parameter of the proposed model and should reflect the assumption that all related genes should have completed their biological reactions; for example, transcription from DNA to mRNA, and translation from mRNA to protein. If we fix the time interval for all genes the FBM, then, is a synchronic Boolean model. If all genes have their time interval defined, the FBM then is an asynchronous Boolean model. The proposed Boolean model, therefore, can be used to reconstruct the missing time steps using either a synchronous scheme or an asynchronous scheme. However, because the results from the asynchronous Boolean model are nondeterministic, we cannot use the reconstructed time series to verify the generated output correctly under the asynchronous Boolean model.

## Conclusions

In this paper, we studied the characteristics of enzyme activation, enzyme inhibition and protein decay as well as the advantages and disadvantages of the conventional Boolean models and then proposed a novel data-driven Boolean model, namely the Fundamental Boolean Model (FBM), to draw insights into gene activation, inhibition, and protein decay. The FBM separated the activation and inhibition functions from conventional Boolean functions, and this separation will facilitate scientists in finding answers to some fundamental questions, such as how a modification of one gene affects other genes at the expression level. We introduced a new data-driven method to infer FBNs. The new method contains two different parts: the first part was to construct an orchard cube to store all precomputed measures for all potential fundamental Boolean functions; the second part was to infer FBNs from the constructed orchard cube by filtering each tree's underground part, based on the criteria discussed previously. Dynamic FBNs could show the significant trajectories of genes to reveal how genes regulate each other over a given period. This new feature could facilitate further research on drug interactions to detect the side effects of the use of a newly proposed drug. The protein decay issue is also a function of the proposed model (Equation 6), and hence, there is three type of links for the FBNs; and this feature makes the networks unique over other Boolean models.

The proposed FBM is a data-driven model, and the FBM functions are extracted from a particular type of data cube. Hence, the knowledge about the connectivity among genes are not needed but can be used to verify the generated result. To prove the concepts of the FBM and FBNs, we implemented an R package called *FBNNet*, which has successfully demonstrated that FBNs can be inferred from time series data. The R package provides a tool to draw FBNs, either in the static mode, as shown in Figure 9, or in the dynamic mode, as illustrated in Figure 11.

The dynamic trajectory of gene activation, gene inhibition and protein decay activities of the attractor 2 is deciphered in Figure 11 confirmed that the proposed FBM could explicitly display the internal connections between genes separated by the connection types of activation, inhibition, and protein decay. We demonstrated the novel concepts of the FBM and the proposed method to infer the proposed FBNs with the mammalian cell cycle networks. The demonstration shows that the method could be used to infer GRNs, with a high degree of accuracy, from the time series data generated from the pre-known Boolean networks via the existing R package, *BoolNet*.

There was a need to search all related genes and to calculate all relevant measures for all associated gene combinations up to some depth, to infer FBNs. This requirement could end with the NP-hard problem (Non-deterministic Polynomial acceptable problems), i.e., there is no known polynomial algorithm so that the time to find a solution grows exponentially with predefined problem size, as mentioned in Liu et al. (2016). However, with the design of the orchard cube, the cost was endurable because the design of the orchard cube was embedded within parallel computations. With the power of computational clouds, even large GRNs can be derived from the method we proposed. Moreover, the construction of the orchard cube was separated from the inference of FBNs; hence, we can use a different strategy to mine the regulatory network without the need to rebuild the cube. Therefore, the computational cost can be split into the construction of an orchard cube and network inference. During the experiments we conducted, we found the construction of an orchard cube consumed the most computational cost. In other words, if we had the orchard cube constructed already, the computational cost for inferring FBNs from the orchard cube is minor and affordable.

## Availability of data and materials

The experimental data and materials are discussed in Appendix B of SI.

## Author contributions

LC, DK, and SS developed the research project, formulated the research questions and designed the paper. LC did all the computing with intellectual inputs from DK in consultation with SS. DK directed the project. LC wrote the first draft with DK and SS critiqued it, and all authors approved the final submitted version.

### Conflict of interest statement

The authors declare that the research was conducted in the absence of any commercial or financial relationships that could be construed as a potential conflict of interest.

## Figures and Tables

**Table:** 

APC	Anaphase-promoting complex
BUC	Bottom-up computation
Cdks	Cyclin-dependent kinases (Cdk1, Cdk2 and so on)
CKI	Cyclin-dependent kinase inhibitor
CycD	Cdk4/6-Cyclin D complex
CycE	Cdk2/Cyclin E complex
CycA	Cdk2/Cyclin A complex
Cdc20	Cell-division cycle protein 20
Cdh1	Epithelial cadherin (E-cadherin), a classical member of the cadherin superfamily
CycB	Cdk1/Cycline B complex
DNA	Deoxyribonucleic acid
E2F	A family of transcription factors (TF) that act as transcriptional regulators of G1–S transcription
GRNs	Genetic regulatory networks
GF	Growth factor
NP	Nondeterministic Polynomial, a computational complexity class
NP-hard	A class of problems in computational complexity (Non-deterministic Polynomial acceptable problems)
p27	A member of Kip/Cip family, a group of CKIs
R	R statistic script programming language
RNA	Ribonucleic acid
Rb	Retinoblastoma protein
SI	Supplementary information
UbcH10	Cancer-related E2 ubiquitin-conjugating enzyme1

**Table:** 

AR	Accurate rate
ER	Error rate
FBM	Fundamental Boolean model
FBNs	Fundamental Boolean networks
MMR	Mismatched rate
PMR	Perfect matched rate

**Table:** 

&	Logical And connector
|	Logical Or connector
!	Logical negation symbol

**Table:** 

¬	A negation operator that changes a Boolean function from TRUE to FALSE or vice versa
×, ∩	Logical And operator
+	logical Or operator
τ	An incremental variable presenting the number of time steps that have been processed
ϑ	The decay period to reflect the fact that the attenuation or enhancement of the expression of mRNA requires time
$\sigma_{i}^{t}$	The Boolean state of gene σ_*i*_ at time t
$\sigma_{i}^{t+1}$	The Boolean state of gene σ_*i*_ at time t + 1.
*l*_*a*_ (i)	The total number of fundamental Boolean functions activating the target gene σ_*i*_.
*l*_*d*_ (i)	The total number of fundamental Boolean functions deactivating the target gene σ_*i*_.
$f_{a_{k}}^{i}$	A fundamental Boolean function of activation
$f_{d_{k}}^{i}$	A fundamental Boolean function of inhibition
*P*〚*x*〛	A Boolean function that takes a uniform distributed random number, μ, and an output of 1 if μ < x and 0 otherwise.
