# Supplementary material for: A Novel Data-Driven Boolean Model for Genetic Regulatory Networks
Source: Front Physiol. 2018 Sep 25;9:1328. doi: 10.3389/fphys.2018.01328 (PMC6167558; doi:10.3389/fphys.2018.01328)
Supplement: Supplementary file 1 [file Data_Sheet_1.zip › SI/Fundamental Boolean Model Supplementary Information Manuscript_clean.docx]

Supplementary Information

# Appendix A

Table 1 Experimental Script for the R package *FBNNet*

require(BoolNet)

require(FBNNet)

#generate BoolNet type of cell cycle network

cellcyclenetwork<-loadNetwork("testthat/others/cellcycle.txt")

trainingseries<-generateTimeSeries(cellcyclenetwork,3000,43)

#reduce the duplicate samples

trainingseries<-FBNDataReduction(trainingseries)

#generate initial states, i.e., get the first state from all samples

startstates<-lapply(trainingseries,function(x)x[,1])

#construct FBM cube, maxK=4, temporal=1

cube<-constructFBNCube(cellcyclenetwork$genes,cellcyclenetwork$genes,trainingseries,4,1,TRUE)

#mine FBN type of network

FBNcellcyclenetwork<-mineFBNNetwork(cube,cellcyclenetwork$genes)

#plot the static FBN type of Network

FBNNetwork.Graph(FBNcellcyclenetwork)

#print out the Network

print(FBNcellcyclenetwork)

#reconstruct the timeseries with initial states with the original time series generated with BoolNet

resultfile<-FBNBenchmark(FBNcellcyclenetwork,NULL,trainingseries,trainingseries,interval=1,temporal=1,FALSE,cube)

#genereate similarity report, i.e., compare the reconstructed time series with the original one

similar<-checkSimilarity(trainingseries,resultfile$FBNResult$reconstructed)

similarreport<-generateSimilarReport(similar)

print("Similar Report")

print(similarreport)

print("Benchmark result")

print(paste("ErrorRate=",resultfile$ErrorRate,sep="",collapse = ""))

print(paste("AccurateRate=",resultfile$AccurateRate,sep="",collapse = ""))

print(paste("MissMatchedRate=",resultfile$MissMatchedRate,sep="",collapse = ""))

print(paste("PerfectMatchedRate=",resultfile$PrfectMatchedRate,sep="",collapse = ""))

#get attractors

genes<-rownames(trainingseries [[1]])

attractor<-searchForAttractors(FBNcellcyclenetwork,startstates,genes)

#display the dynamic trajectory of the attactor 2

FBNNetwork.Graph.DrawAttractor(FBNcellcyclenetwork,attractor,2)

# Appendix B

Many new algorithms can be verified using the simulated datasets derived from several known regulatory networks, and the results can be compared with other known regulatory networks. In this paper, we proposed to use the mammalian cell cycle network, to generate test data to prove the concept of the proposed Fundamental Boolean Model and Networks. The mammalian cell cycle network demonstrated in (Hopfensitz, Mussel et al. 2013). To generate the experimental data, firstly, we used the command loadNetwork from *BoolNet* to load the cell cycle network specified in the text files: cellcycle.txt, as shown in Table 2. Secondly, we use the method generateTimeSeries of *BoolNet* to generate 1024 noiseless sample data with 43 time steps for the cell cycle network, with all default settings, i.e., the parameter type is synchronous, the parameter noiseLevel is 0, and the parameter perturbations is 0. Each sample contains the same 10 mammalian cell cycle genes, i.e., CycD, Rb, E2F, CycE, CycA, p27, Cdc20, Cdh1, UbcH10, and CycB, as the study conducted by Hopfensitz et al.(2013). The generated 1024 sample data are the dataset used for this experiment. Each sample contains 43 time steps in sequence. All the initial states of the 1024 samples are unique containing the complete combination of $2^{10}$ changes. Hence, the number of genes that are expressed in each sample is variant.

.

Table 2 Mammalian Cell Cycle Network

| **File Name: cellcycle.txt, Network Type: mammalian cell cycle network** |
| --- |
| targets, factors  CycD, CycD  Rb, (! CycA & ! CycB & ! CycD & ! CycE) \| (p27 & ! CycB & ! CycD)  E2F, (! Rb & ! CycA & ! CycB) \| (p27 & ! Rb & ! CycB)  CycE, (E2F & ! Rb)  CycA, (E2F & ! Rb & ! Cdc20 & ! (Cdh1 & UbcH10)) \| (CycA & ! Rb & ! Cdc20 & ! (Cdh1 & UbcH10))  p27, (! CycD & ! CycE & ! CycA & ! CycB) \| (p27 & ! (CycE & CycA) & ! CycB &! CycD)  Cdc20, CycB  Cdh1,(! CycA & ! CycB) \| (Cdc20) \| (p27 & ! CycB)  UbcH10, ! Cdh1 \| (Cdh1 & UbcH10 & (Cdc20 \| CycA \| CycB))  CycB, ! Cdc20 & ! Cdh1 |
| **File Name: example.txt, Network Type: Example Network** |
| targets, factors  Gene1, Gene1  Gene2, Gene1 & Gene5 & !Gene4  Gene3, Gene3  Gene4, Gene3 & !(Gene1 & Gene5)  Gene5,!Gene2 |


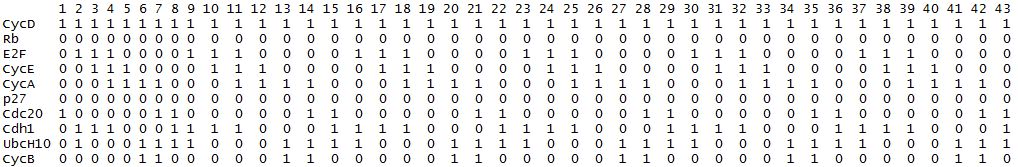


Figure 1 One of the generated time series data for the Cell cycle network.

The R package *FBNNet* also contains the experiment result conducted in this paper for the demonstration of the mammalian cell cycle network, as shown in Table 3.

Table 3 Experimental Script for loading the experiment result conducted in this paper

loadExperimentData<-function()

{

#to remove all variables we can use the following command

# rm(list=ls(all=TRUE))

print("load synchronous training series data -> synchronoustrainingseries. \n\n")

load("synchronoustrainingseries.RDATA",envir = parent.frame())

print("load synchronous cellcycle cube -> synchronous_cellcycle_cube. \n\n")

load("synchronous_cellcycle_cube.RDATA",envir = parent.frame())

print("load asynchronous training series data -> asynchronoustrainingseries. \n\n")

load("asynchronoustrainingseries.RDATA",envir = parent.frame())

print("load asynchronous cellcycle cube -> asynchronous_cellcycle_cube. \n\n")

load("asynchronous_cellcycle_cube.RDATA",envir = parent.frame())

print("load leukeamia data -> leukeamia. \n\n")

load("leukeamia.RDATA",envir = parent.frame())

print("load cellcycle genes -> cellcyclegenes \n\n")

load("cellcyclegenes.RDATA")

genes<<-rownames(synchronoustrainingseries[[1]])

synfbnnetwork<<-mineFBNNetwork(synchronous_cellcycle_cube,genes)

asynfbnnetwork<<-mineFBNNetwork(asynchronous_cellcycle_cube,genes)

startstates<<-lapply(synchronoustrainingseries,function(x)x[,1])

attractor<<-searchForAttractors(synfbnnetwork,startstates,genes)

}

# Appendix C

Table 4 An Example of Fundamental Boolean Network

| Fundamental Boolean Network with 5 genes  Genes involved:  Gene1, Gene2, Gene3, Gene4, Gene5  Multiple Transition Functions for Gene1 with decay value = 1:  Gene1_1_Activator: Gene1 = Gene1 (Confidence: 1, TimeStep: 1)  Gene1_2_Inhibitor: Gene1 = !Gene1 (Confidence: 1, TimeStep: 1)  Multiple Transition Functions for Gene2 with decay value = 1:  Gene2_1_Activator: Gene2 = Gene1&!Gene4&Gene5 (Confidence: 1, TimeStep: 1)  Gene2_2_Inhibitor: Gene2 = !Gene1 (Confidence: 1, TimeStep: 1)  Gene2_3_Inhibitor: Gene2 = Gene4 (Confidence: 1, TimeStep: 1)  Gene2_4_Inhibitor: Gene2 = !Gene5 (Confidence: 1, TimeStep: 1)  Multiple Transition Functions for Gene3 with decay value = 1:  Gene3_1_Activator: Gene3 = Gene3 (Confidence: 1, TimeStep: 1)  Gene3_2_Inhibitor: Gene3 = !Gene3 (Confidence: 1, TimeStep: 1)  Multiple Transition Functions for Gene4 with decay value = 1:  Gene4_1_Activator: Gene4 = !Gene1&Gene3 (Confidence: 1, TimeStep: 1)  Gene4_2_Activator: Gene4 = Gene3&!Gene5 (Confidence: 1, TimeStep: 1)  Gene4_3_Inhibitor: Gene4 = !Gene3 (Confidence: 1, TimeStep: 1)  Gene4_4_Inhibitor: Gene4 = Gene1&Gene5 (Confidence: 1, TimeStep: 1)  Multiple Transition Functions for Gene5 with decay value = 1:  Gene5_1_Activator: Gene5 = !Gene2 (Confidence: 1, TimeStep: 1)  Gene5_2_Inhibitor: Gene5 = Gene2 (Confidence: 1, TimeStep: 1) |
| --- |

## FBN Measurement Example

### An example of the calculation of a Fundamental Boolean Function

As given in Table 4, the wiring diagram of the Example network represents the dependencies between activation and inhibition, wherein the expression level of gene 4 at time *t+1* not only depends on the value of the activation rule associated with the expression level of gene 3 at time *t* but also depends on the value of the inhibition rule related to the expression level of genes 1 and 5 at time *t*. Hence, the model for gene 4 to be activated or inhibited at next time step is outlined below:

$${Gene}_{4}^{t+1}=(f_{decay}({gene}_{4}^{t},1)+P\left⟦ C_{a_{1}}^{4}\left\lfloor f_{a_{1}}^{4}\left( {!gene}_{1}^{t}\&{gene}_{3}^{t} \right) \right\rfloor\right⟧+P\left⟦ C_{a_{2}}^{4}\left\lfloor f_{a_{2}}^{4}\left( {gene}_{3}^{t}\&{!gene}_{5}^{t} \right) \right\rfloor\right⟧) \times\neg(P\left⟦ C_{d_{1}}^{4}\left\lfloor f_{d_{1}}^{4}\left( {!gene}_{3}^{t} \right) \right\rfloor\right⟧+ P\left⟦ C_{d_{2}}^{4}\left\lfloor f_{d_{2}}^{4}\left( {gene}_{1}^{t}\& g{ene}_{5}^{t} \right) \right\rfloor\right⟧)$$

If the gene state at time *t* is: $\mathrm{gene}_{1}$=1, g$\mathrm{ene}_{3}$=1, $\mathrm{gene}_{4}$=1, $\mathrm{gene}_{5}$=1 and the protein decay is 1, the above formula, then, is transferred to:

$${Gene}_{4}^{t+1}=(1+P\left⟦ C_{a_{1}}^{4}\left\lfloor0 \right\rfloor\right⟧+P\left⟦ C_{a_{2}}^{4}\left\lfloor0 \right\rfloor\right⟧) \times\neg(P\left⟦ C_{d_{1}}^{4}\lfloor0\rfloor\right⟧+P\left⟦ C_{d_{2}}^{4}\lfloor1\rfloor\right⟧)$$

Therefore, the final result of ${Gene}_{4}$ at time *t* +1 can be calculated if $C_{d_{2}}^{4}\left\lfloor1 \right\rfloor$ =1 and hence, $P\left⟦ 1 \right⟧=1$ ($P\left⟦ 0 \right⟧=0)$:

$${Gene}_{4}^{t+1}=\left( 1+0+0 \right)\times\neg\left( 0+1 \right)=1\times\neg\left( 1 \right)=1\times0=0$$

The final result of the above formula is 0, which indicates that $\mathrm{gene}_{4}$ at time *t* +1 is inhibited.

### Examples of Other Measures

Suppose we have three samples for the genes: CycD, p27, CycE, and E2F, as shown in Table 5.

Table 5 Sample data for precomputed measures

|  | Sample 1 | | | | Sample 2 | | | | Sample 3 | | | |
| --- | --- | --- | --- | --- | --- | --- | --- | --- | --- | --- | --- | --- |
| **Timepoint**  **Genes** | **1** | **2** | **3** | **4** | **1** | **2** | **3** | **4** | **1** | **2** | **3** | **4** |
| CycD | 1 | 0 | 1 | 0 | 1 | 0 | 0 | 1 | 1 | 1 | 1 | 1 |
| p27 | 1 | 1 | 1 | 0 | 1 | 1 | 1 | 0 | 0 | 1 | 1 | 1 |
| CycE | 1 | 0 | 1 | 0 | 0 | 0 | 0 | 0 | 0 | 0 | 0 | 0 |
| E2F | 1 | 1 | 1 | 1 | 1 | 1 | 1 | 1 | 1 | 1 | 1 | 1 |

#### Confidence Measures:

We use the data shown in Table 5 to calculate the confidence measure on the hypothesis that the gene *CycD* is activated by the activation of *p27*, i.e., the regulatory function $f_{a_{j}}^{i}: {p27}^{1}\to{CycD}^{1}$, so we use the formulae in Eq.(5.a). First, we calculate the value for $p\left( A_{CycD}^{j}\left( t \right)=1 \right)$, where $A_{CycD}^{j}\left( t \right)$: *p27*=1, at time *t* = {1, 2, 3}. As shown in Table 5, the value of $p\left( A_{CycD}^{j}\left( t \right)=1 \right)$ is equal to 0.8889 (8 divided by 9). Now, we calculate the values for $p\left( A_{CycD}^{j}\left( t \right)=1\cap\sigma_{i}^{t+1}=1 \right)$, where $\sigma_{i}^{t+1}$: *CycD*(t+1), at time *t* = {2, 3, 4}. The value for $p\left( A_{CycD}^{j}\left( t \right)=1\cap\sigma_{i}^{t+1}=1 \right)$ is 0.4444 (4 divided by 9). Therefore, the confidence measure for the hypothesis is:

$$C_{a_{j}}^{CycD}\left\lfloor f_{a_{j}}^{CycD}\left( p27\left( t \right) \right) \right\rfloor=p\left( CycD(t+1)=1 | p27\left( t \right)=1 \right)=\frac{0.4444}{0.8889} \approx0.5$$

#### Confidence Counter Measure:

Secondly, to calculate the confidence counter measure on the same hypothesis as the above example, we can use the formulae in Eq.(8.a). As shown in Table 5, the value of $p\left( CycD(t)=1 \right)$ is equal to 0.6667 (6 divided by 9) at time *t* = {1, 2, 3} and the values for $p\left( A_{CycD}^{j}\left( t+1 \right)=1\cap\sigma_{i}^{t}=1 \right)$, where $\sigma_{i}^{t}$: *CycD*(t), at time *t* = {2, 3, 4} is 0.5556 (5 divided by 9). Therefore, the confidence counter measure is:

$${C\forall}_{a_{j}}^{CycD}\left\lfloor f_{a_{j}}^{CycD}\left( p27\left( t+1 \right) \right) \right\rfloor=p\left( p27\left( t+1 \right)=1 | CycD(t)=1 \right)=\frac{0.5556}{0.6667} \approx0.83$$

#### Support Measures:

Using the same data, as shown in Table 5, to calculate the support measure on the same hypothesis, we use the formulae in Eq.(9.a). First, we calculate the value for $\aleph$, which is 9 in this case. Secondly, we calculate the values for $count\left( p27\left( t \right)=1 \cap CycD(t+1)=1 \right)$ and the value is 4. Therefore, the support measure for the hypothesis is:

$$Support of activation=\frac{4}{9}\approx0.4444$$

#### Conditional Causality Test:

To calculate the conditional causality test measure with the same hypothesis as the above examples, we can use the formulae in Eq.(10) and the values of the confidence measure and confidence counter measure calculated from the above examples to obtain the value of the conditional causality test.

$$Conditional causality test=\frac{0.5}{0.83}\approx0.6$$

Hence, the hypothesis should be rejected because the value of the conditional causality test is less than 1.

#### Entropy and mutual information:

For example, we have two gene states: gene *A* (1, 0, 1, 0, 1) and gene *B* (1, 1, 1, 0, 1), and we would like to know whether or not gene *B* is determined by gene *A*. To resolve this requirement, we need to calculate M(*B, A*) by subtracting H(*B|A*) from H(*B*). The following outlines the main calculations:

Because

$$H\left( B|A \right)=-\left( \frac{3}{3}\times{log}_{2} \left( \frac{3}{3} \right)+0\times{log}_{2} \left( 0 \right)+ \right)=-1\times0=0$$

Hence

$$M\left( B,A \right)=H\left( B \right)-H\left( B | A \right)=H\left( B \right)-0=H\left( B \right)$$

Therefore, we can claim that gene *B* is determined by gene *A* because of $M\left( B,A \right)$=H(B).

# Appendix D

## A Literature **Review** on Boolean modeling

The Boolean model (also called switching model) is a simple, discrete dynamic model without the need to consider the effects at the intermediate levels (Tušek and Kurtanjek 2012) and is one of the most interest in the field of GRNs (Shmulevich and Dougherty 2005, Li, Zhang et al. 2007, Wang, Saadatpour et al. 2012, Ouyang, Fang et al. 2014, Zhiyuan, Simone et al. 2014). Boolean network models do not need information about kinetic parameters and; hence, they are powerful in qualitatively describing large-scale system dynamics (Wang, Saadatpour et al. 2012). The biological basis of the Boolean network is that cells exhibit switch-like behavior during the regulation of their functional states. The switch-like behavior ensures the movement of a cell from one state to another (Shmulevich, Dougherty et al. 2002a, Shmulevich and Dougherty 2005, Tušek and Kurtanjek 2012). Scientists can physically turn Boolean models into electronic circuits so we can study the rich dynamics of Boolean networks using mathematical and signal processing theory (Yufei 2009).

Kauffman (Kauffman 1969, Kauffman, Peterson et al. 2003) proposed a concept of Boolean networks in 1969 and, since then, the concept has been intensively used for modeling gene regulation (Kauffman 1969, Thomas 1973, Bornholdt 2005, Bornholdt 2008, Davidich and Bornholdt 2008). Boolean networks only have two distinct values: *On* and *Off* (1 and 0) and are discrete, dynamical and consistent. According to the original definition of Kauffman, the Boolean network is defined as a graph *G (V, E)*, annotated with a collection of states $X=\left\{ x_{i} \right|i=1,\ldots,n\}$, together with a set of Boolean functions:

$$F=\left\{ f_{i} | i=1,\ldots,k \right\}, f_{i}:\left\{ 0,1 \right\}\to\{0,1\}$$

where each node$v_{i}$ has been associated with a Boolean function, with inputs of the states of the nodes connected to$v_{i}$. The state of node $v_{i}$at the time *t* is expressed as$v_{i}(t)$. Hence, the state of that node at time *t+1* is given by:

$$v_{i}\left( t+1 \right)= f_{i}(x_{i1}, x_{i2},\ldots, x_{ik})$$

where $x_{ij}$ is the state of the nodes connected to$v_{i}$.

Boolean models have been categorized into two main types of schema based on the similarity of timescales for all biological events (Gershenson 2004, Wang, Saadatpour et al. 2012). The two categories are synchronous scheme (also called deterministic systems) and asynchronous scheme. In synchronous systems, all variables are assumed to have similar timescales and will be simultaneously updated, i.e., one unit will update all components simultaneously:

$$\sigma_{i}^{t+1}=B_{i}(\sigma_{i_{1}}^{t}, \sigma_{i_{2}}^{t}, . . . , \sigma_{i_{k_{i}}}^{t})$$

where $B=\{B_{1}, B_{2}, \ldots, B_{n}\}$ is a set of Boolean functions (Wang, Saadatpour et al. 2012) and $\sigma_{i_{1}}, \sigma_{i_{2}}, . . . , \sigma_{i_{k_{i}}}$ is a set of Boolean variables with size k. In contrast, all variables will be updated non-simultaneously in asynchronous schemes if most of the timescales of biological actions are different, i.e., each component will be updated at their own time unit.

$$\sigma_{i}^{*}=B_{i}(\sigma_{i_{1}}, \sigma_{i_{2}}, . . . , \sigma_{i_{k_{i}}})$$

where the asterisk denotes the variable $\sigma_{i}^{*}$ (i = 1, 2,..., n) is derived from the set of inputs $\sigma_{i_{1}}, \sigma_{i_{2}}, . . . , \sigma_{i_{k_{i}}}$. The inputs can be the gene states from the current or previous time point (Wang, Saadatpour et al. 2012).

Both types of schemes can be mapped to a directed graph *G(V, E)*, where the node set $V=\{v_{1},v_{2},\ldots,v_{n}\}$ corresponds to the Boolean variables with size *N*, and the edge set *E* matches the Boolean functions in the model. Each edge has a direction with a sign indicating how the input node affects the target node (positively or negatively) (Wang, Saadatpour et al. 2012). A vector ($\sigma_{1(t)}, \sigma_{2(t)}, \ldots,\sigma_{i\left( t \right)}, \ldots,\sigma_{n\left( t \right)}$) is referred to as the state of the system at time *t* (Wang, Saadatpour et al. 2012). The *i*th vector variable $\sigma_{i\left( t \right)}$denotes the state of node $v_{i}$ at time *t*. Each node can be linked to a gene, a protein, and a metabolite in order to elucidate the dynamics of biological systems using Boolean networks.

Synchronous Boolean networks have the assumption that the state of a gene at a given time step is influenced by the state of a subset of genes in the network at the previous time step. A drawback of the synchronous dynamics is that it does not allow the temporal separation of multiple regulatory activity changes (Faure, Naldi et al. 2006). Another drawback is that the synchronous Boolean network model cannot measure differences in the speed of signal propagation because no two cells have the same properties; hence, this results in differences in the rates of signal propagation between cells in the context of biological systems (Hwang and Lee 2010). The most popular synchronous Boolean models are the Random Boolean Networks (Gershenson 2002, Mc 2002, Kauffman, Peterson et al. 2003, Shmulevich, Lahdesmaki et al. 2003, Gershenson 2004, Samuelsson 2006, Drossel 2007, Lynch 2007), Temporal Boolean Networks (Silvescu and Honavar 2001), Probabilistic Boolean Networks (Shmulevich, Dougherty et al. 2002, Shmulevich, Dougherty et al. 2002, Shmulevich, Dougherty et al. 2002a, Shmulevich, Dougherty et al. 2002b, Dougherty and Shmulevich 2003, Shmulevich, Gluhovsky et al. 2003, Shmulevich and Dougherty 2005, Harri, Harri et al. 2006), Threshold Boolean Networks (Higa, Andrade et al. 2013), Stochastic Boolean Networks (Wang, Yang et al. 2008, Liang and Han 2012), Petri Boolean Models (Chaouiya 2007, Berestovsky, Zhou et al. 2013) and Switching Boolean Networks(Hwang and Lee 2010).

In the model of Random Boolean Network (RBN), random Boolean functions control the state of each node by randomly selecting Boolean functions from the $2^{2^{k}}$possible *K* input. The randomly selected Boolean functions are then kept fixed afterwards. After studying the dynamics of these RBNs, Kauffman claimed that the existence of a phase transition in an RBN of size N depends on the value of the parameter K (Kauffman 1969, Kauffman, Peterson et al. 2003, Albert 2004). If K is more than 2, there are approximately *N/e* (e is the base of the natural logarithm) possible cycles of scales that exponentially lengthen to N. If the K is equal to 2, both the number and duration of the limit cycles are approximately the square root of the network size N, i.e., $\sqrt{N}$ (Albert 2004).

Silvescu et al. extended the works of (Liang, Fuhrman et al. 1998, Akutsu, Miyano et al. 1999) on the inference of Boolean networks to handle multiple time steps and introduced the temporal Boolean networks (Silvescu and Honavar 2001). The temporal Boolean networks fundamentally renovated the Boolean networks from a Markov(1) to a Markov(T) model where T is the length of the time window during which a gene can influence another gene (Silvescu and Honavar 2001). Silvescu and Honavar demonstrated that the temporal Boolean networks could be inferred from time series data. The central obstruction of applying temporal Boolean networks to a real system is the lack of sufficiently large datasets (Silvescu and Honavar 2001).

Threshold Boolean networks have a Boolean function for each gene, so the output value depends only on the sum of its input signals (Hwang and Lee 2010). The limitation of a threshold Boolean network is that it entirely relies on the completion of network information. If the network is incomplete, the result is a modeling anomaly (Hwang and Lee 2010).

The asynchronous update only allows the update of one gene or component at a random time, resulting in a nondeterministic representation of the dynamics (Siebert 2011). A drawback of the asynchronous scheme is that the resulting state transition graph is very complex and encompasses many incompatible or unrealistic pathways (Faure, Naldi et al. 2006). There are also some modifications proposed for asynchronous Boolean models, such as non-deterministic asynchronous Boolean networks and deterministic asynchronous Boolean networks (Gershenson 2004).

Models that combine synchronous and asynchronous transitions can illustrate the flexibility of the combination of different updating assumptions (Faure, Naldi et al. 2006). Berezovsky et al. proposed an integrated hybrid model (IHM) that combines Petri nets and Boolean networks to model integrated cellular networks. The hybrid model can be applied to three main cellular biochemical processes: signal transduction, transcription regulation and metabolism (Chaouiya 2007, Berestovsky, Zhou et al. 2013).

Some conventional approaches were proposed to infer Boolean functions: *Symbolic* approaches (Yoon 2005, Batt, de Jong et al. 2008, Langmead and Jha 2008), *REVEAL* approaches (Liang, Fuhrman et al. 1998), the *Best-Fit Extension* algorithm, *gene FAt* (Zheng, Yang et al. 2013) and *Chi-square test* (Kim, Lee et al. 2007). The Reverse Engineering Algorithm (REVEAL) (Liang, Fuhrman et al. 1998) was later extended to allow for multiple discrete states as well as to let the current state depend as a window of previous states (Hecker, Lambeck et al. 2009). Mussel et al. proposed a tool named the BoolNet R package (Mussel, Hopfensitz et al. 2010) for generating, reconstructing and analysing Boolean networks from time series using the Best-Fit Extension (Shmulevich, Yli-Harja et al. 2002) and REVEAL algorithms (Liang, Fuhrman et al. 1998). The tool provides methods to identify the attractors of synchronous, asynchronous and probabilistic Boolean networks. The package has been demonstrated a tutorial by (Hopfensitz, Mussel et al. 2013). The advantage of the package over another existing tool, such as GINsim (Gonzalez, Naldi et al. 2006), BooleanNet (Albert, Thakar et al. 2008) and BN/PBN toolbox in Matlab, was that it supported all three network types.

With the facilities of the emerged Boolean modeling tools, Boolean networks have been successfully applied to yeast (Kauffman, Peterson et al. 2003, Li, Long et al. 2004, Davidich and Bornholdt 2008, Kazemzadeh, Cvijovic et al. 2012), flower morphogenesis of wall cress, *Arabidopsis thaliana (Espinosa-Soto, Padilla-Longoria et al. 2004)*, *Drosophila melanogaster* (Sanchez and Thieffry 2001, Albert and Othmer 2003, Ghysen and Thomas 2003), MOMP regulation (Tokar, Turcan et al. 2013), the mammalian cell cycle (Faure, Naldi et al. 2006, Ruz, Goles et al. 2014), the light- and carbon-signalling pathways (Thum, Shasha et al. 2003), apoptosis networks (Mai and Liu 2009, Schlatter, Schmich et al. 2009, Kazemzadeh, Cvijovic et al. 2012, Schleich and Lavrik 2013), the hepatocyte signal networks (Schlatter, Philippi et al. 2012), NF-kappaB and IL-6 mediated by miRNA (Xue, Xia et al. 2013) and leukaemia(Saez-Rodriguez, Simeoni et al. 2007, Wittmann, Krumsiek et al. 2009, Hwang and Lee 2010, Saadatpour, Wang et al. 2011, Saadatpour, Albert et al. 2013, Zanudo and Albert 2013, Campbell and Albert 2014).

In this successful analysis, Davidich and Bornholdt predicted the biological cell cycle sequence of fission yeast using a Boolean model, with 47 kinetic constants that were necessary for the ODE (ordinary differential equations) approach dropped, and it was assumed that the biochemical network was functioning in a parameter-insensitive way (Davidich and Bornholdt 2008). Faure et al. extended the software GINsim and studied the dynamics of a Boolean model for the control of the mammalian cell cycle, with synchronous, asynchronous or hybrid treatment of concurrent transitions (Faure, Naldi et al. 2006). Moreover, the signal transduction network for abscisic acid has been proven to induce stomatal closure (Li, Assmann et al. 2006).

Boolean networks have now been applied to various samples. However, the restriction of short time series data still discourages researchers from using the Boolean model to analyse real human genomic data, such as the GC-regulated gene data.

# References

Akutsu, T., S. Miyano and S. Kuhara (1999). "Identification of genetic networks from a small number of gene expression patterns under the Boolean network model." Pac Symp Biocomput: 17-28.

Albert, I., J. Thakar, S. Li, R. Zhang and R. Albert (2008). "Boolean network simulations for life scientists." Source Code Biol Med **3**: 16.

Albert, R. (2004). "Boolean Modeling of Genetic Regulatory Networks." Lect. Notes Phys **650**: 459-481.

Albert, R. and H. G. Othmer (2003). "The topology of the regulatory interactions predicts the expression pattern of the segment polarity genes in Drosophila melanogaster." Journal of Theoretical Biology **223**(1): 1-18.

Batt, G., H. de Jong, M. Page and J. Geiselmann (2008). "Symbolic reachability analysis of genetic regulatory networks using discrete abstractions." Automatica **44**(4): 982-989.

Berestovsky, N., W. Zhou, D. Nagrath and L. Nakhleh (2013). "Modeling integrated cellular machinery using hybrid Petri-Boolean networks." PLoS Comput Biol **9**(11): e1003306.

Bornholdt, S. (2005). "Systems biology. less is more in modeling large genetic networks." Science **310**(5747): 449-451.

Bornholdt, S. (2008). "Boolean network models of cellular regulation: prospects and limitations." J R Soc Interface **5 Suppl 1**: S85-94.

Campbell, C. and R. Albert (2014). "Stabilization of perturbed Boolean network attractors through compensatory interactions." BMC Syst Biol **8**: 53.

Chaouiya, C. (2007). "Petri net modelling of biological networks." Brief Bioinform **8**(4): 210-219.

Davidich, M. and S. Bornholdt (2008). "The transition from differential equations to Boolean networks: a case study in simplifying a regulatory network model." J Theor Biol **255**(3): 269-277.

Davidich, M. I. and S. Bornholdt (2008). "Boolean network model predicts cell cycle sequence of fission yeast." PLoS One **3**(2): e1672.

Dougherty, E. R. and I. Shmulevich (2003). "Mappings between probabilistic Boolean networks." Signal Processing **83**(4): 799-809.

Drossel, B. (2007). "Random Boolean Networks."

Espinosa-Soto, C., P. Padilla-Longoria and E. R. Alvarez-Buylla (2004). "A gene regulatory network model for cell-fate determination during Arabidopsis thaliana flower development that is robust and recovers experimental gene expression profiles." Plant Cell **16**(11): 2923-2939.

Faure, A., A. Naldi, C. Chaouiya and D. Thieffry (2006). "Dynamical analysis of a generic Boolean model for the control of the mammalian cell cycle." Bioinformatics **22**(14): e124-131.

Gershenson, C. (2002). Classification of random Boolean networks. Proceedings of the 8th International Conference on Artificial Life. R. K. e. a. e. Standish, MIT Press**:** 1-8.

Gershenson, C. (2004). "Introduction to random Boolean networks." in Proceedings of the Workshops and Tutorials of the Ninth International Conference on the Simulation and Synthesis of Living Systems (ALife IX), Boston, USA, ed. by M. Bedau, P. Husbands, T. Hutton, S. Kumar, H. Suzuki: 160-173.

Ghysen, A. and R. Thomas (2003). "The formation of sense organs in Drosophila: a logical approach." Bioessays **25**(8): 802-807.

Gonzalez, A. G., A. Naldi, L. Sanchez, D. Thieffry and C. Chaouiya (2006). "GINsim: a software suite for the qualitative modelling, simulation and analysis of regulatory networks." Biosystems **84**(2): 91-100.

Harri, L., L. Harri, S. Ilya and S. Ilya (2006). "Relationships between probabilistic Boolean networks and dynamic Bayesian networks as models of gene regulatory networks." Signal Processing **86**(4): Signal Processing.

Hecker, M., S. Lambeck, S. Toepfer, E. van Someren and R. Guthke (2009). "Gene regulatory network inference: data integration in dynamic models-a review." Biosystems **96**(1): 86-103.

Higa, C. H., T. P. Andrade and R. F. Hashimoto (2013). "Growing seed genes from time series data and thresholded Boolean networks with perturbation." IEEE/ACM Trans Comput Biol Bioinform **10**(1): 37-49.

Hopfensitz, M., C. Mussel, M. Maucher and H. A. Kestler (2013). "Attractors in Boolean networks: a tutorial." Computational Statistics **28**(1): 19-36.

Hwang, W. and D. Lee (2010). "Cell Signaling Dynamics Analysis in Leukemia with Switching Boolean Networks." Computational Systems Biology **13**: 168-175.

Kauffman, S., C. Peterson, B. Samuelsson and C. Troein (2003). "Random Boolean network models and the yeast transcriptional network." Proc Natl Acad Sci U S A **100**(25): 14796-14799.

Kauffman, S. A. (1969). "Metabolic stability and epigenesis in randomly constructed genetic nets." J Theor Biol **22**(3): 437-467.

Kazemzadeh, L., M. Cvijovic and D. Petranovic (2012). "Boolean model of yeast apoptosis as a tool to study yeast and human apoptotic regulations." Front Physiol **3**: 446.

Kim, H., J. K. Lee and T. Park (2007). "Boolean networks using the Chi-square test for inferring large-scale gene regulatory networks." BMC Bioinformatics **8**: 37.

Langmead, C. J. and S. K. Jha (2008). Symbolic approaches for finding control strategies in Boolean networks. Proceedings of the sixth Asia-Pacific Bioinformatics Conference.

Li, F., T. Long, Y. Lu, Q. Ouyang and C. Tang (2004). "The yeast cell-cycle network is robustly designed." Proc Natl Acad Sci U S A **101**(14): 4781-4786.

Li, P., C. Zhang, E. J. Perkins, P. Gong and Y. Deng (2007). "Comparison of probabilistic Boolean network and dynamic Bayesian network approaches for inferring gene regulatory networks." BMC Bioinformatics **8 Suppl 7**: S13.

Li, S., S. M. Assmann and R. Albert (2006). "Predicting essential components of signal transduction networks: a dynamic model of guard cell abscisic acid signaling." PLoS Biol **4**(10): e312.

Liang, J. and J. Han (2012). "Stochastic Boolean networks: an efficient approach to modeling gene regulatory networks." BMC Syst Biol **6**: 113.

Liang, S., S. Fuhrman and R. Somogyi (1998). "Reveal, a general reverse engineering algorithm for inference of genetic network architectures." Pac Symp Biocomput: 18-29.

Lynch, J. F. (2007). "Dynamics of random boolean networks." Current Developments in Mathematical Biology **38**: 15-38.

Mai, Z. and H. Liu (2009). "Boolean network-based analysis of the apoptosis network: irreversible apoptosis and stable surviving." J Theor Biol **259**(4): 760-769.

Mc, A. J. (2002). "Random Boolean Networks and Evolutionary Game Theory." Philosophy of Science Assoc.18th Biennial Mtg.

Mussel, C., M. Hopfensitz and H. A. Kestler (2010). "BoolNet - an R package for generation, reconstruction and analysis of Boolean networks." Bioinformatics **26**(10): 1378-1380.

Ouyang, H., J. Fang, L. Shen, E. R. Dougherty and W. Liu (2014). "Learning restricted Boolean network model by time-series data." EURASIP J Bioinform Syst Biol **2014**(1): 10.

Ruz, G. A., E. Goles, M. Montalva and G. B. Fogel (2014). "Dynamical and topological robustness of the mammalian cell cycle network: a reverse engineering approach." Biosystems **115**: 23-32.

Saadatpour, A., R. Albert and T. C. Reluga (2013). "A reduction method for Boolean network models proven to conserve attractors." Siam Journal on Applied Dynamical Systems **12**(4): 1997-2011.

Saadatpour, A., R. S. Wang, A. Liao, X. Liu, T. P. Loughran, I. Albert and R. Albert (2011). "Dynamical and structural analysis of a T cell survival network identifies novel candidate therapeutic targets for large granular lymphocyte leukemia." PLoS Comput Biol **7**(11): e1002267.

Saez-Rodriguez, J., L. Simeoni, J. A. Lindquist, R. Hemenway, U. Bommhardt, B. Arndt, U. U. Haus, R. Weismantel, E. D. Gilles, S. Klamt and B. Schraven (2007). "A logical model provides insights into T cell receptor signaling." PLoS Comput Biol **3**(8): e163.

Samuelsson, B. (2006). Dynamics in random Boolean networks. Doctor of Philosophy, Lund University.

Sanchez, L. and D. Thieffry (2001). "A logical analysis of the Drosophila gap-gene system." J Theor Biol **211**(2): 115-141.

Schlatter, R., N. Philippi, G. Wangorsch, R. Pick, O. Sawodny, C. Borner, J. Timmer, M. Ederer and T. Dandekar (2012). "Integration of Boolean models exemplified on hepatocyte signal transduction." Brief Bioinform **13**(3): 365-376.

Schlatter, R., K. Schmich, I. Avalos Vizcarra, P. Scheurich, T. Sauter, C. Borner, M. Ederer, I. Merfort and O. Sawodny (2009). "ON/OFF and beyond - a Boolean model of apoptosis." PLoS Comput Biol **5**(12): e1000595.

Schleich, K. and I. N. Lavrik (2013). "Mathematical modeling of apoptosis." Cell Commun Signal **11**(1): 44.

Shmulevich, I. and E. Dougherty (2005). Modeling genetic regulatory networks with probabilistic Boolean networks. New York, USA, Hindawi.

Shmulevich, I., E. R. Dougherty, S. Kim and W. Zhang (2002a). "Probabilistic Boolean networks: a rule-based uncertainty model for gene regulatory networks." Bioinformatics **18**(2): 261-274.

Shmulevich, I., E. R. Dougherty and W. Mang (2002b). "From Boolean to probabilistic boolean networks as models of genetic regulatory networks." Proceedings of the IEEE **90**(11): 1778-1792.

Shmulevich, I., E. R. Dougherty and W. Zhang (2002). "Control of stationary behavior in probabilistic Boolean networks by means of structural intervention." Journal of Biological Systems **10**(4): 431-445.

Shmulevich, I., E. R. Dougherty and W. Zhang (2002). "Gene perturbation and intervention in probabilistic Boolean networks." Bioinformatics **18**(10): 1319-1331.

Shmulevich, I., I. Gluhovsky, R. F. Hashimoto, E. R. Dougherty and W. Zhang (2003). "Steady-state analysis of genetic regulatory networks modelled by probabilistic Boolean networks." Comp Funct Genomics **4**(6): 601-608.

Shmulevich, I., H. Lahdesmaki, E. R. Dougherty, J. Astola and W. Zhang (2003). "The role of certain post classes in Boolean network models of genetic networks." Proc Natl Acad Sci U S A **100**(19): 10734-10739.

Shmulevich, I., O. Yli-Harja and J. Astola (2002). Inference of genetic regulatory networks under the best-fit extension paradigm Proceedings of the IEEE-EURASIP workshop on nonlinear signal and image processing (NSIP-01).

Siebert, H. (2011). "Logic-based modeling in system biology."

Silvescu, A. and V. Honavar (2001). "Temporal Boolean network models of genetic networks and their inference from gene expression time series." Complex Systems **13**(61-78).

Thomas, R. (1973). "Boolean formalization of genetic control circuits." J Theor Biol **42**(3): 563-585.

Thum, K. E., D. E. Shasha, L. V. Lejay and G. M. Coruzzi (2003). "Light- and carbon-signaling pathways. Modeling circuits of interactions." Plant Physiol **132**(2): 440-452.

Tokar, T., Z. Turcan and J. Ulicny (2013). "Boolean network-based model of the Bcl-2 family mediated MOMP regulation." Theoretical Biology and Medical Modelling.

Tušek, A. and Ž. Kurtanjek (2012). Mathematical modelling of gene regulatory networks. London, SE1 9SG, UNITED KINGDOM, InTech.

Wang, R. S., A. Saadatpour and R. Albert (2012). "Boolean modeling in systems biology: an overview of methodology and applications." Phys Biol **9**(5): 055001.

Wang, Z., F. Yang, D. W. Ho, S. Swift, A. Tucker and X. Liu (2008). "Stochastic dynamic modeling of short gene expression time-series data." IEEE Trans Nanobioscience **7**(1): 44-55.

Wittmann, D. M., J. Krumsiek, J. Saez-Rodriguez, D. A. Lauffenburger, S. Klamt and F. J. Theis (2009). "Transforming Boolean models to continuous models: methodology and application to T-cell receptor signaling." BMC Syst Biol **3**: 98.

Xue, X., W. Xia and H. Wenzhong (2013). "A modeled dynamic regulatory network of NF-kappaB and IL-6 mediated by miRNA." Biosystems **114**(3): 214-218.

Yoon, S. (2005). Genomic data mining enhanced by symbolic manipulation of Boolean functions. Doctor of Philosophy, Stanford University.

Yufei, X. (2009). "A tutorial on analysis and simulation of Boolean gene regulatory network models." Current Genomics **10**: 511-525.

Zanudo, J. G. and R. Albert (2013). "An effective network reduction approach to find the dynamical repertoire of discrete dynamic networks." Chaos **23**(2): 025111.

Zheng, D., G. Yang, X. Li, Z. Wang, F. Liu and L. He (2013). "An efficient algorithm for computing attractors of synchronous and asynchronous Boolean networks." PLoS One **8**(4): e60593.

Zhiyuan, L., B. Simone, Z. Zhaoyang and T. Chao (2014). "Generic properties of random gene regulatory networks." Quantitative Biology **1**(4): 253–260.

# Figure Legends

[Figure 1 One of the generated time series data for the Cell cycle network. 2](#_Toc524688903)

# Table Legends

[Table 1 Experimental Script for the R package *FBNNet* 1](#_Toc524688904)

[Table 2 Mammalian Cell Cycle Network 2](#_Toc524688905)

[Table 3 Experimental Script for loading the experiment result conducted in this paper 3](#_Toc524688906)

[Table 4 An Example of Fundamental Boolean Network 4](#_Toc524688907)

[Table 5 Sample data for precomputed measures 5](#_Toc524688908)
