# Supplementary figures and images for: A Novel Data-Driven Boolean Model for Genetic Regulatory Networks
Source: Front Physiol. 2018 Sep 25;9:1328. doi: 10.3389/fphys.2018.01328 (PMC6167558; doi:10.3389/fphys.2018.01328)

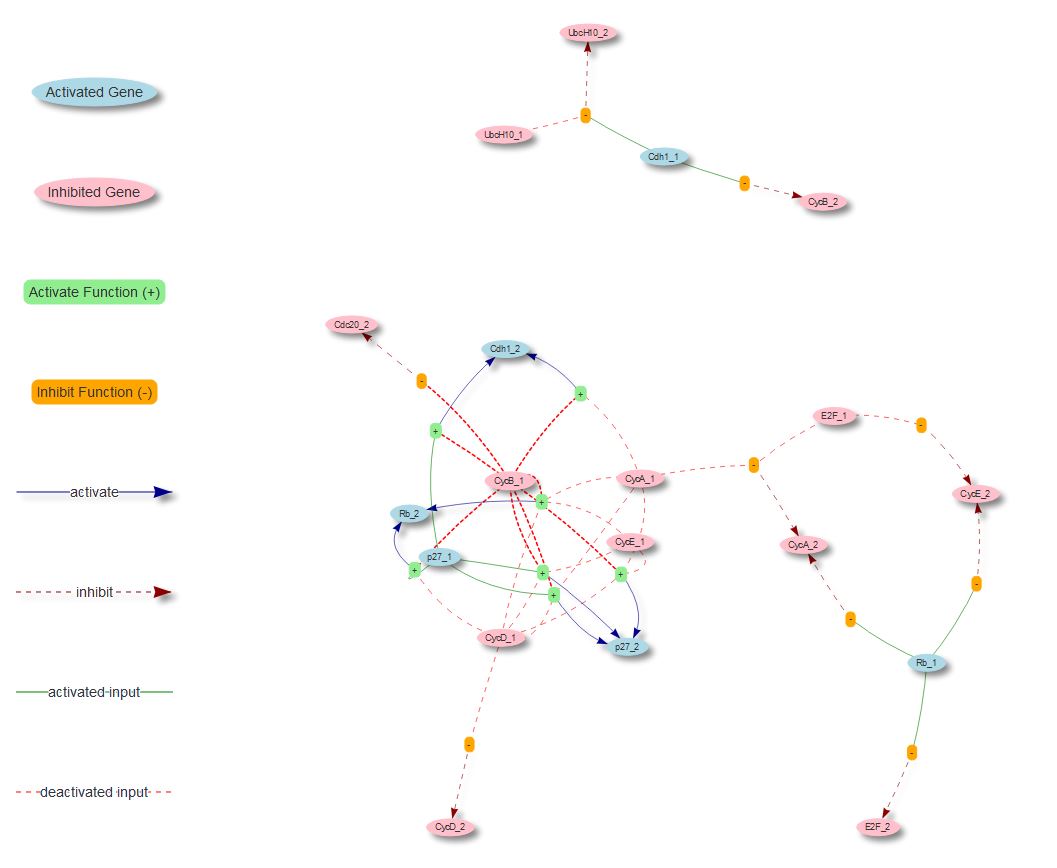

Supplement: Supplementary file 1 [file Data_Sheet_1.zip › SI/revisited Images/Attractor1.JPG]

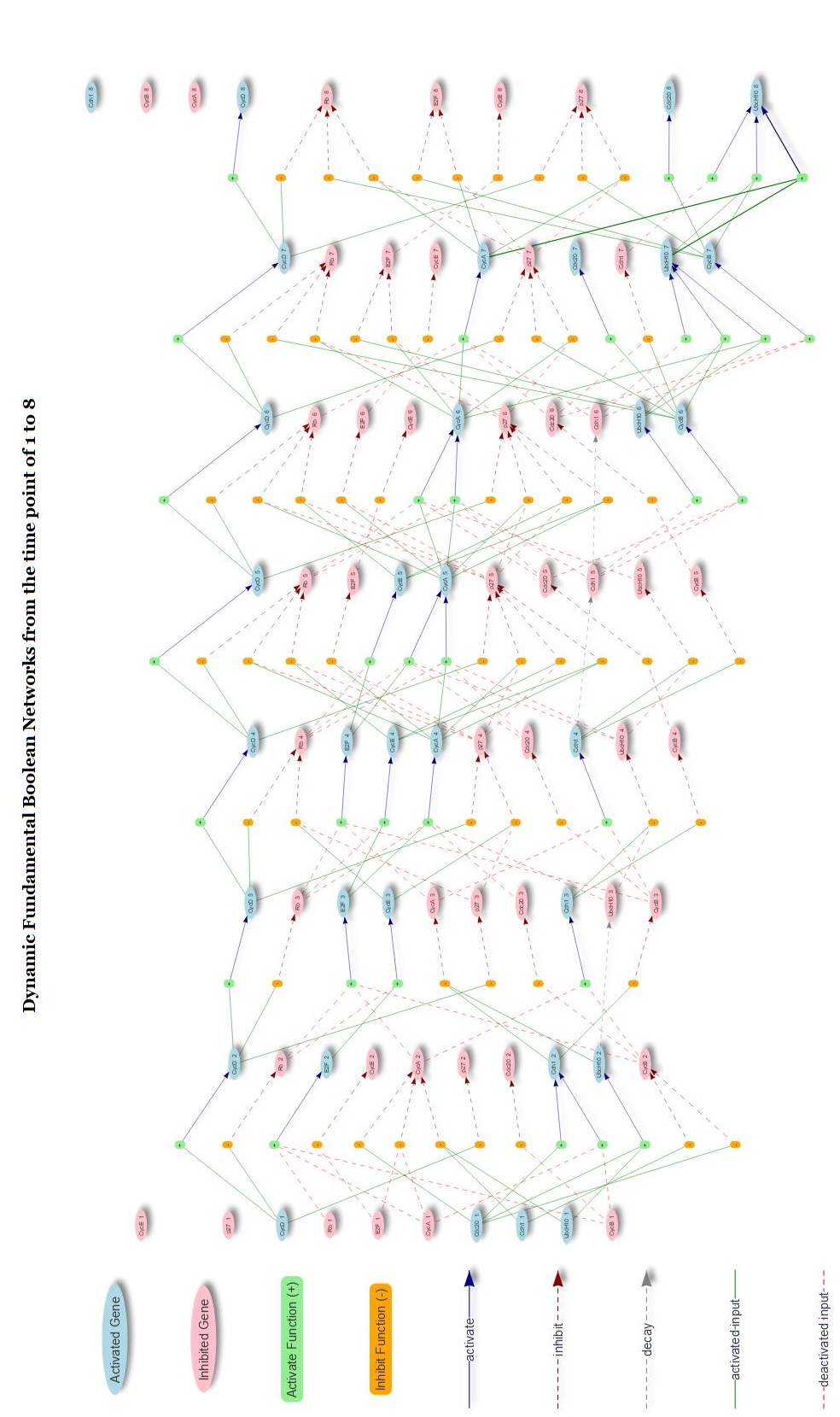

Supplement: Supplementary file 1 [file Data_Sheet_1.zip › SI/revisited Images/attractor2.JPG]

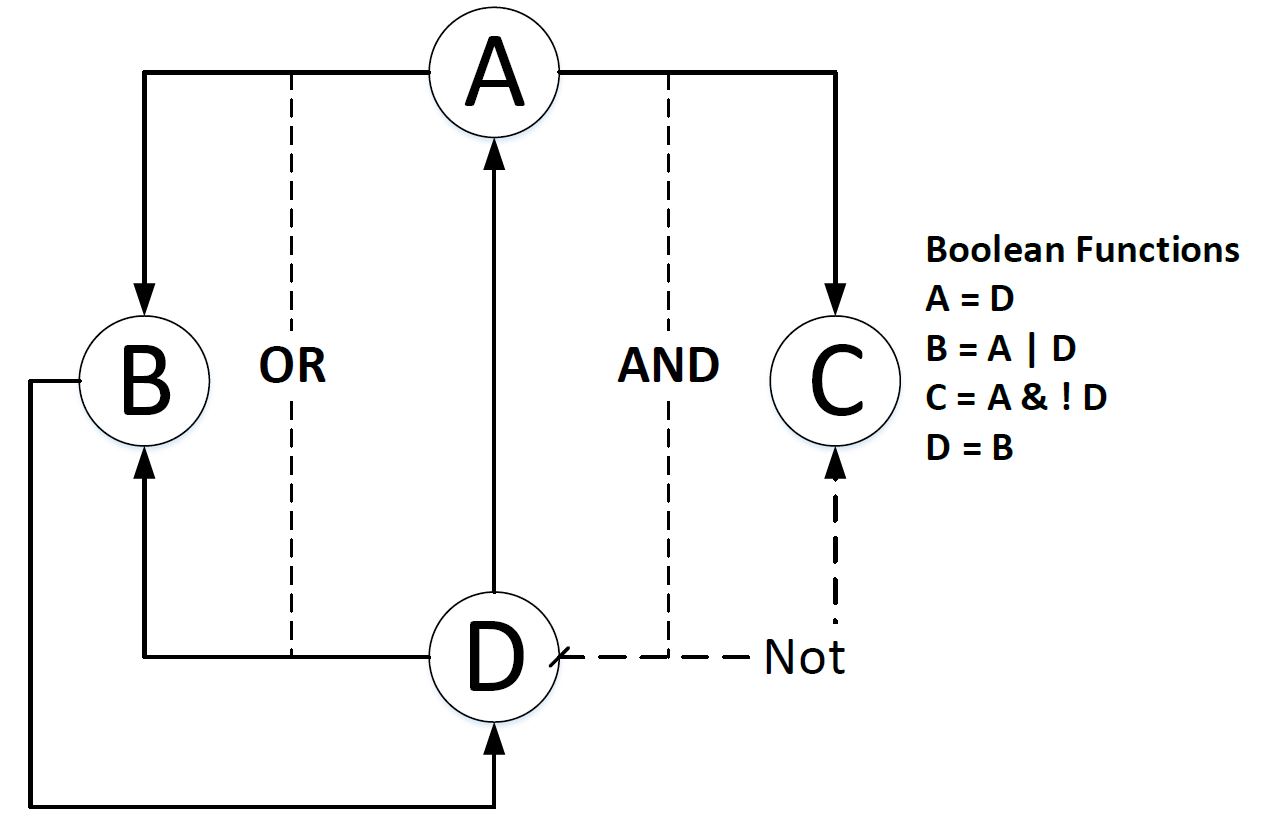

Supplement: Supplementary file 1 [file Data_Sheet_1.zip › SI/revisited Images/Boolean Function samples.JPG]

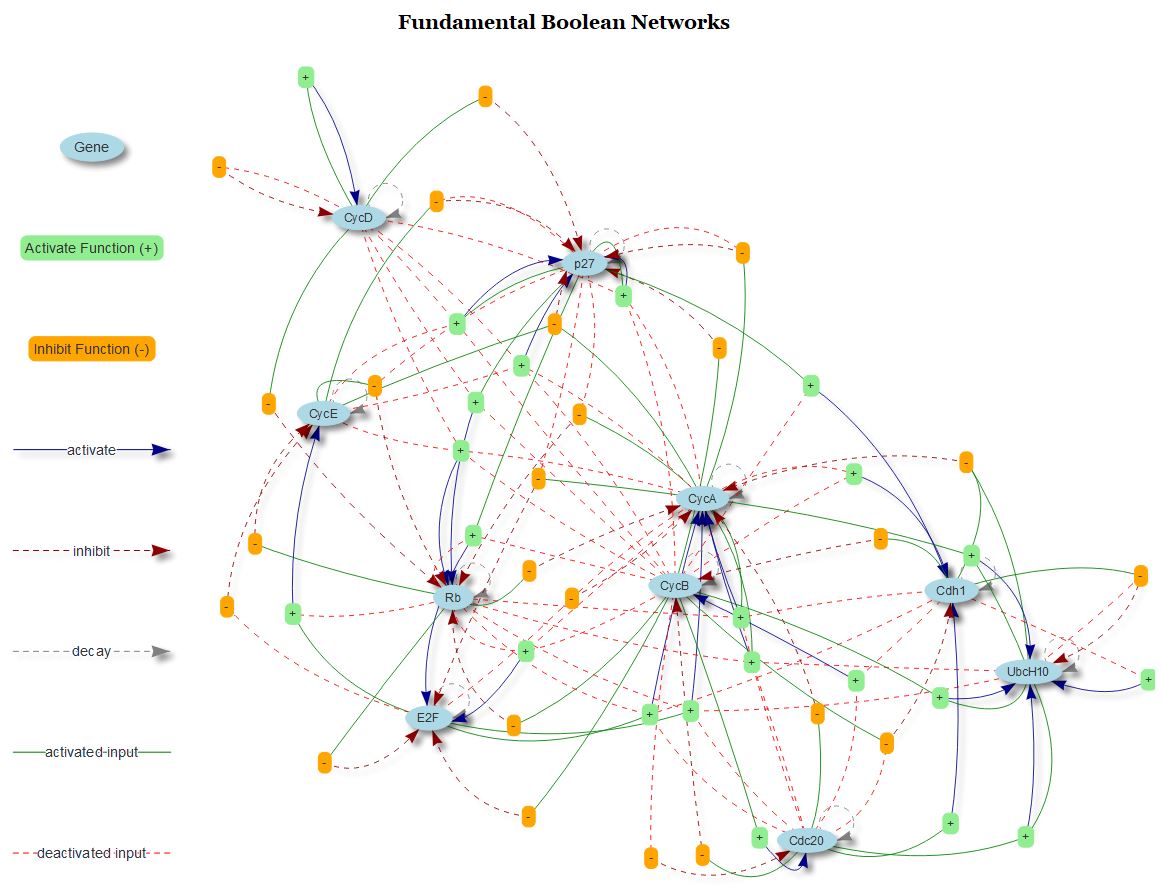

Supplement: Supplementary file 1 [file Data_Sheet_1.zip › SI/revisited Images/Cellcycle FBNs.JPG]

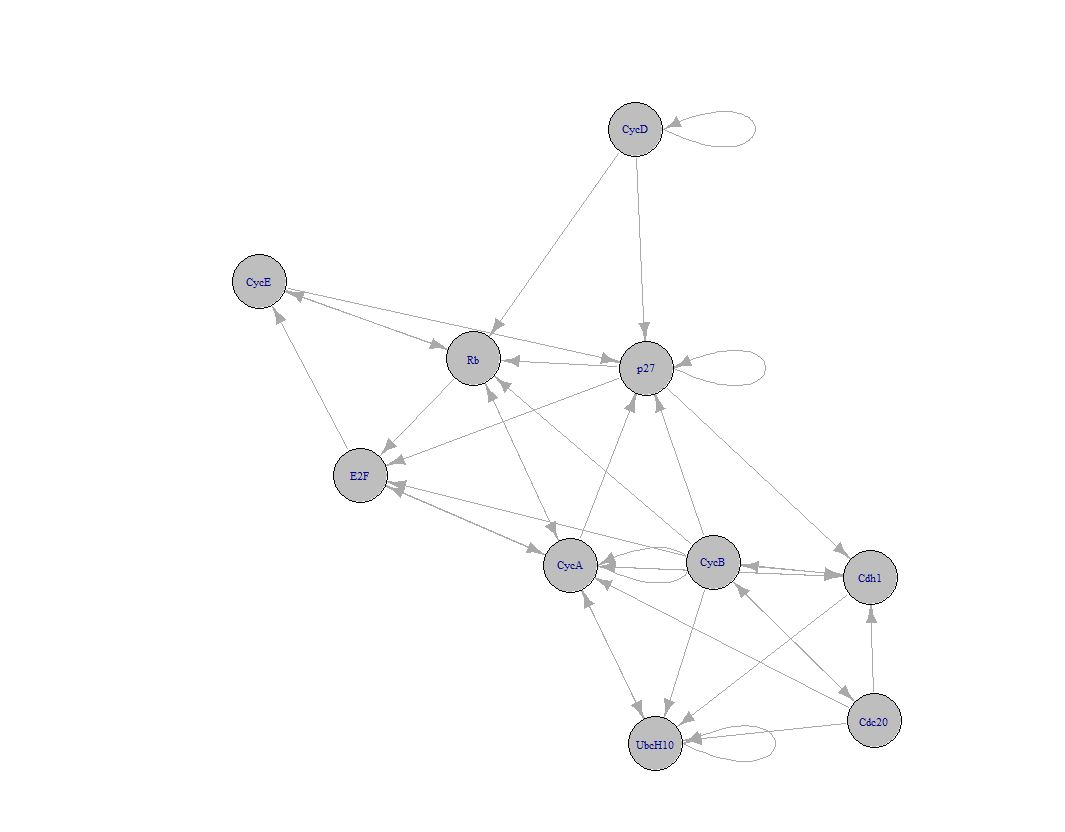

Supplement: Supplementary file 1 [file Data_Sheet_1.zip › SI/revisited Images/Cellcycle Network via BoolNet.JPG]

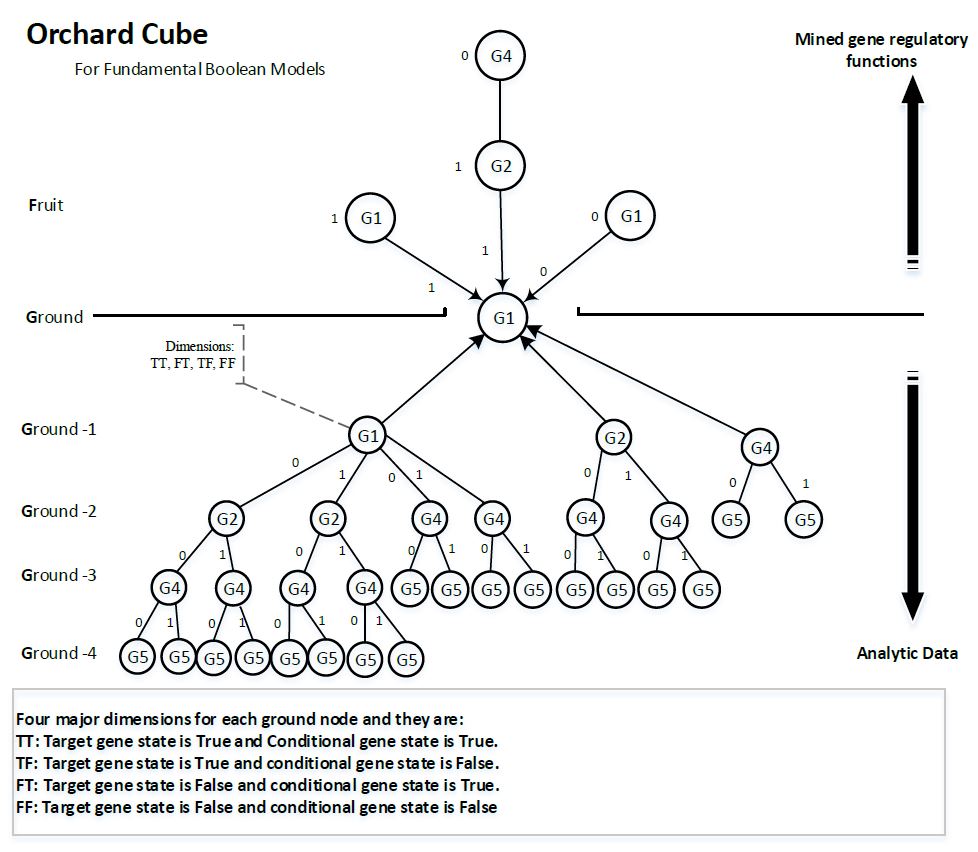

Supplement: Supplementary file 1 [file Data_Sheet_1.zip › SI/revisited Images/Cube.JPG]

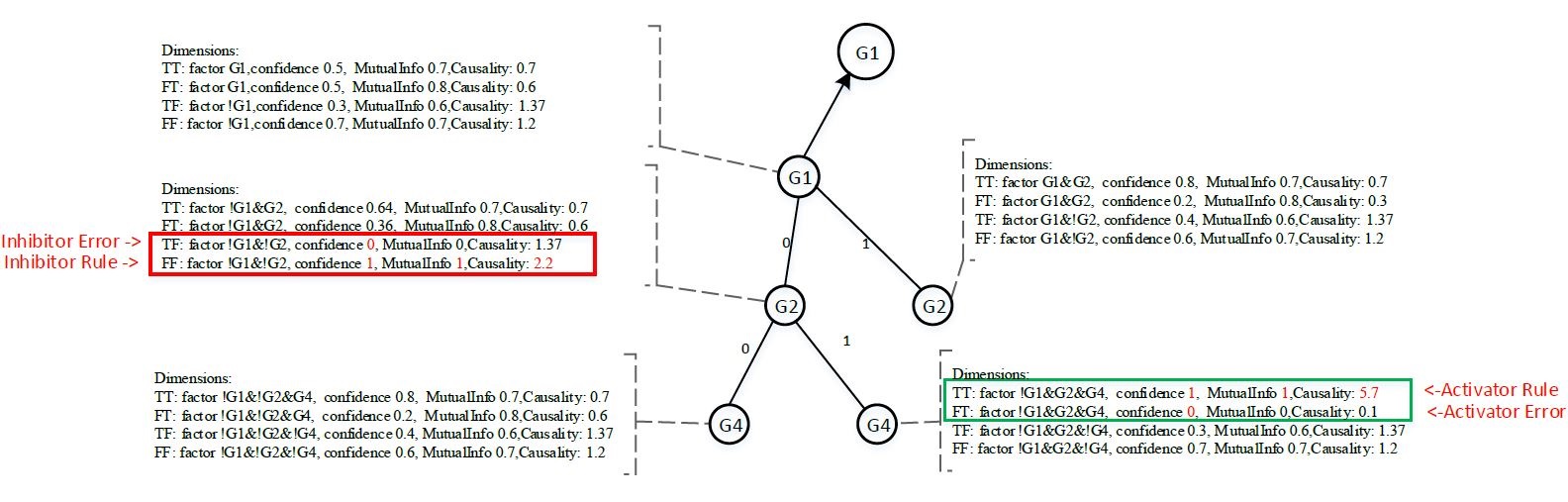

Supplement: Supplementary file 1 [file Data_Sheet_1.zip › SI/revisited Images/Cube2.JPG]

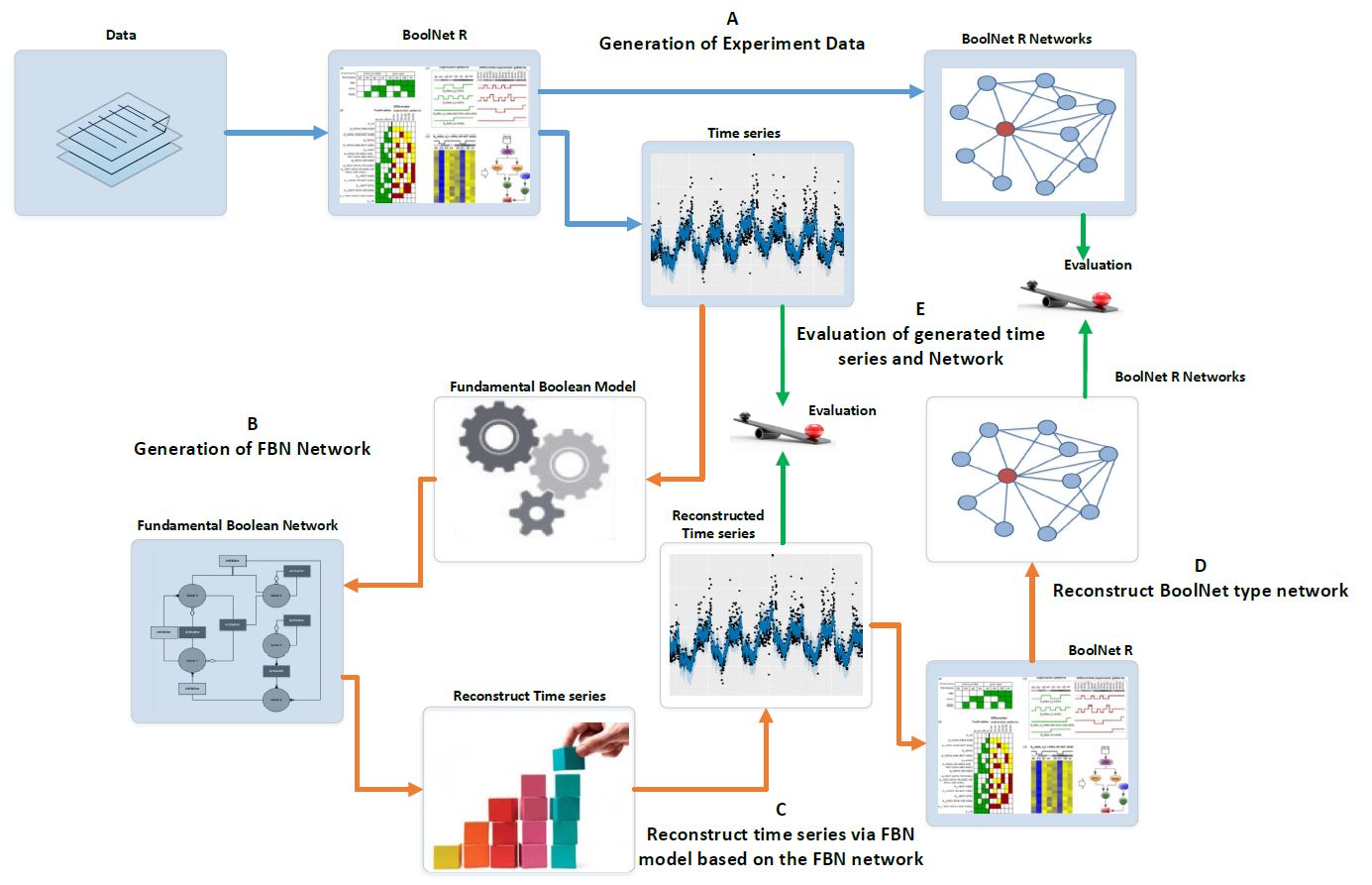

Supplement: Supplementary file 1 [file Data_Sheet_1.zip › SI/revisited Images/Evaluation1.JPG]

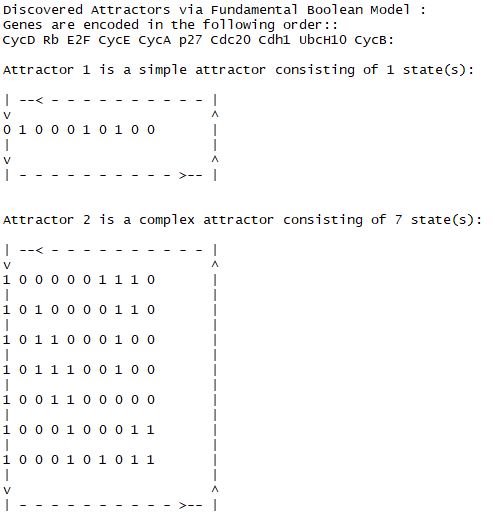

Supplement: Supplementary file 1 [file Data_Sheet_1.zip › SI/revisited Images/FBMAttractors_cellcycle.jpg]

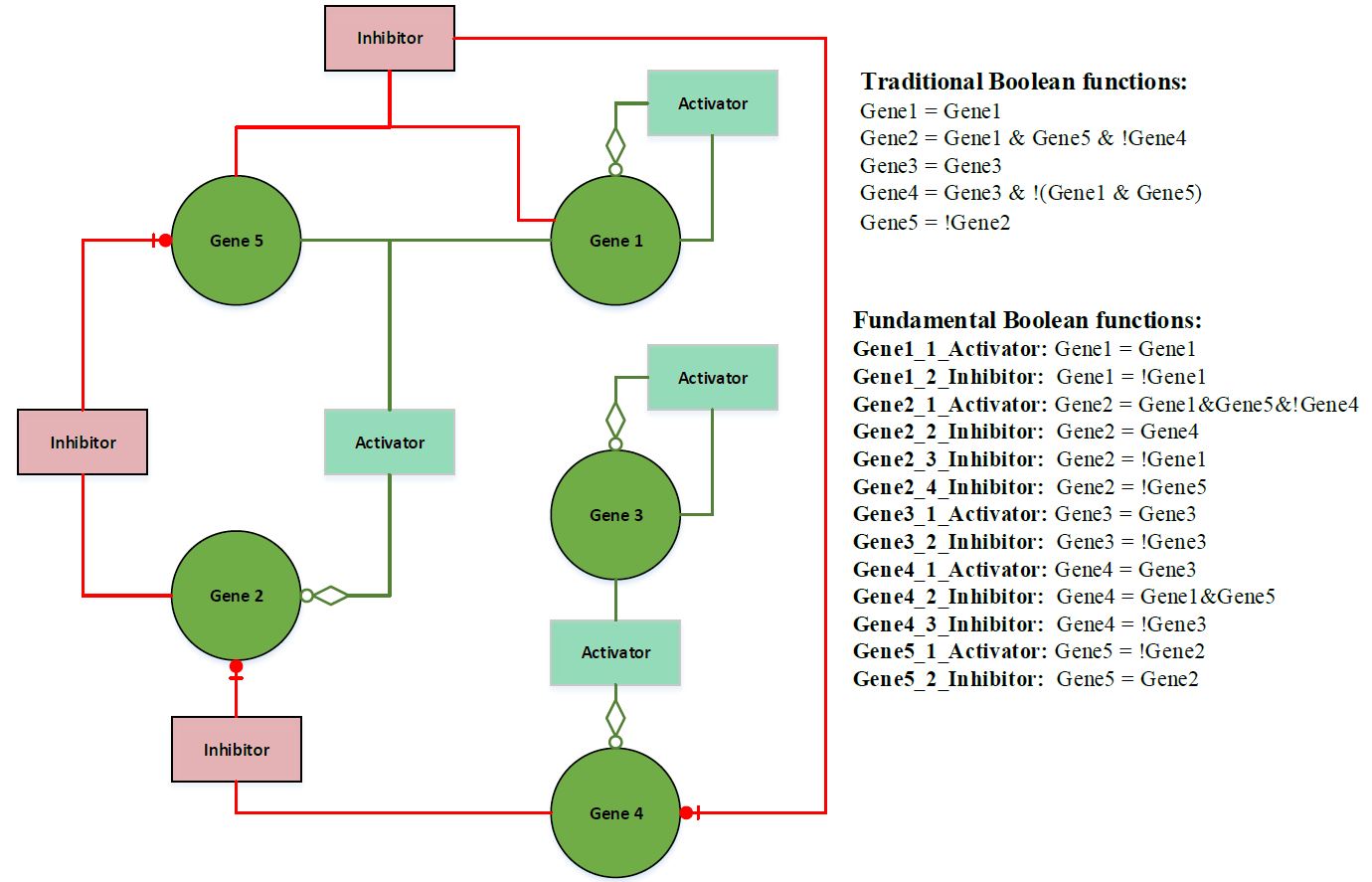

Supplement: Supplementary file 1 [file Data_Sheet_1.zip › SI/revisited Images/FundamentalBoolean.JPG]

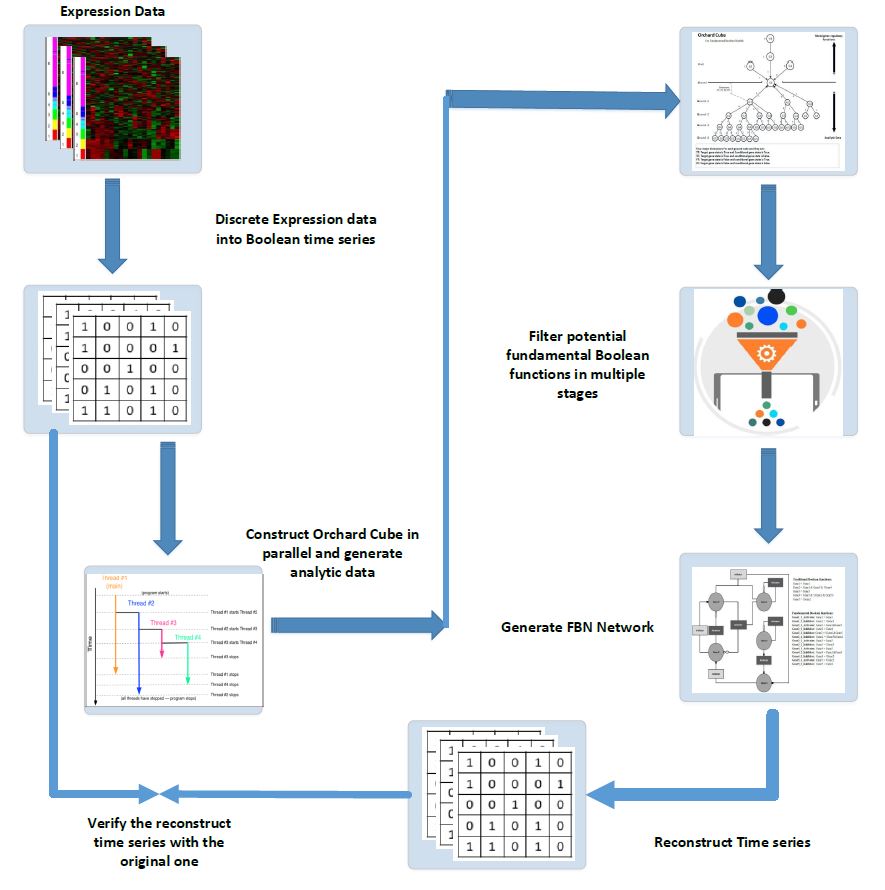

Supplement: Supplementary file 1 [file Data_Sheet_1.zip › SI/revisited Images/MainFlowChart.JPG]

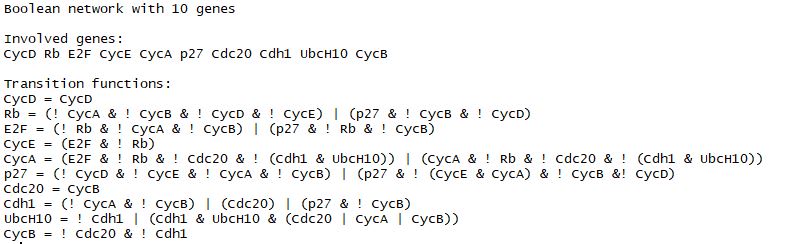

Supplement: Supplementary file 1 [file Data_Sheet_1.zip › SI/revisited Images/OroginalCellcycleNetwork.JPG]

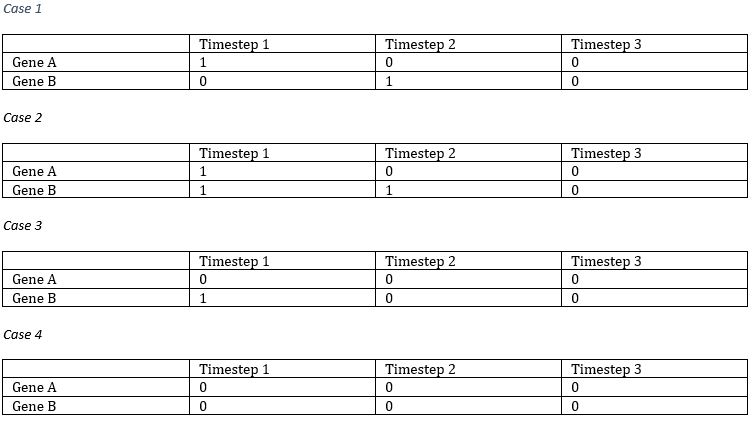

Supplement: Supplementary file 1 [file Data_Sheet_1.zip › SI/revisited Images/Simulation of equilibrium.JPG]
